# The brown fat-enriched exosomal miR-206-3p attenuates hepatic lipogenesis by decreasing pentose phosphate pathway

**DOI:** 10.1093/lifemeta/loaf028

**Published:** 2025-07-12

**Authors:** Li-Jie Yang, Qiu-kai Tang, Lei Wang, Yan-Jue Song, Zhen-Yu Xu, Xi-Ni Ma, Yang Liu, Shu-Wen Qian, Qi-Qun Tang, Yan Tang

**Affiliations:** Key Laboratory of Metabolism and Molecular Medicine, Ministry of Education, Department of Biochemistry and Molecular Biology of School of Basic Medical Sciences and Department of Endocrinology and Metabolism of Zhongshan Hospital, Fudan University, Shanghai 200032, China; Clinical Laboratory, Zhejiang Sian International Hospital, Jiaxing, Zhejiang 314031, China; Key Laboratory of Metabolism and Molecular Medicine, Ministry of Education, Department of Biochemistry and Molecular Biology of School of Basic Medical Sciences and Department of Endocrinology and Metabolism of Zhongshan Hospital, Fudan University, Shanghai 200032, China; Key Laboratory of Metabolism and Molecular Medicine, Ministry of Education, Department of Biochemistry and Molecular Biology of School of Basic Medical Sciences and Department of Endocrinology and Metabolism of Zhongshan Hospital, Fudan University, Shanghai 200032, China; Key Laboratory of Metabolism and Molecular Medicine, Ministry of Education, Department of Biochemistry and Molecular Biology of School of Basic Medical Sciences and Department of Endocrinology and Metabolism of Zhongshan Hospital, Fudan University, Shanghai 200032, China; Key Laboratory of Metabolism and Molecular Medicine, Ministry of Education, Department of Biochemistry and Molecular Biology of School of Basic Medical Sciences and Department of Endocrinology and Metabolism of Zhongshan Hospital, Fudan University, Shanghai 200032, China; Key Laboratory of Metabolism and Molecular Medicine, Ministry of Education, Department of Biochemistry and Molecular Biology of School of Basic Medical Sciences and Department of Endocrinology and Metabolism of Zhongshan Hospital, Fudan University, Shanghai 200032, China; Key Laboratory of Metabolism and Molecular Medicine, Ministry of Education, Department of Biochemistry and Molecular Biology of School of Basic Medical Sciences and Department of Endocrinology and Metabolism of Zhongshan Hospital, Fudan University, Shanghai 200032, China; Key Laboratory of Metabolism and Molecular Medicine, Ministry of Education, Department of Biochemistry and Molecular Biology of School of Basic Medical Sciences and Department of Endocrinology and Metabolism of Zhongshan Hospital, Fudan University, Shanghai 200032, China; Key Laboratory of Metabolism and Molecular Medicine, Ministry of Education, Department of Biochemistry and Molecular Biology of School of Basic Medical Sciences and Department of Endocrinology and Metabolism of Zhongshan Hospital, Fudan University, Shanghai 200032, China

**Keywords:** brown adipose tissue, exosomes, miR-206-3p, pentose phosphate pathway, MAFLD

## Abstract

Brown adipose tissue (BAT) orchestrates interorgan crosstalk through secreted mediators, including proteins, lipids, and exosomal microRNAs (miRNAs). However, the precise molecular identities and functional contributions of these mediators remain elusive. In this study, we isolated exosomes from BAT and conducted miRNA sequencing, identifying miR-206-3p as a previously unrecognized exosomal miRNA with the potential to alleviate metabolic dysfunction-associated fatty liver disease (MAFLD). *In vivo*, adipose-specific knockout of miR-206-3p in mice exacerbated obesity-induced MAFLD, glucose intolerance, insulin resistance, and impaired energy expenditure. Mechanistically, BAT-derived miR-206-3p is selectively packaged into exosomes via a BAT-specific “exo motif” and transported to the liver, where it targets the 3′ untranslated regions (3′-UTRs) of glucose-6-phosphate dehydrogenase (*G6pd*) and transketolase (*Tkt*), which are key enzymes in the pentose phosphate pathway (PPP). The PPP generates nicotinamide adenine dinucleotide phosphate (NADPH) and ribulose-5-phosphate (Ru-5-P) to support lipogenesis and nucleotide synthesis. miR-206-3p modulates these processes by decreasing NADPH production to inhibit hepatic lipid synthesis and increasing Ru-5-P availability to promote cell proliferation. Notably, obese individuals exhibit reduced serum exosomal miR-206-3p alongside upregulated hepatic PPP enzymes. Our study reveals that BAT-derived exosomal miR-206-3p serves as a mediator of BAT−liver crosstalk, suggesting its potential as a therapeutic target for obesity-related disorders, particularly MAFLD.

## Introduction

The global obesity epidemic is a major driver of metabolic dysfunction-associated fatty liver disease (MAFLD) [1]. Currently affecting approximately 30% of the global population, with annual prevalence increasing by 1% [2], MAFLD remains without approved pharmacological treatments despite decades of research [3], creating an urgent need for effective therapies. The pathophysiological complexity of MAFLD arises from dysregulated adipose–liver crosstalk [4, 5], wherein insulin resistance impairs adipose function, disrupting lipoprotein metabolism and promoting hepatic steatosis via ectopic lipid deposition [6]. Notably, thermogenic (brown and beige) adipocytes counteract metabolic dysfunction through uncoupling protein 1 (UCP1)-mediated adaptive thermogenesis [7]. Although brown adipose tissue ( BAT) activation ameliorates obesity and hepatic steatosis [8–10], it maintains glucose uptake even in UCP1-deficient mice [11, 12], indicating functions beyond UCP1-dependent thermogenesis.

BAT functions as a major secretory organ, releasing batokines including lipids, proteins, and miRNAs [13, 14]. Transplantation of BAT from lean mice into obese mice markedly ameliorates hepatic steatosis while improving insulin sensitivity and glucose homeostasis [15–17]. Although several BAT-derived proteins (e.g. neuregulin 4 (Nrg4), phospholipid transfer protein (PLTP), interleukin-6 (IL-6), and insulin-like growth factor 1 (IGF1)) exhibit hepatoprotective effects [15, 18], the full spectrum of protective secretory factors remains to be characterized. Studies using Dicer knockout mice confirm BAT as a significant source of circulating exosomal miRNAs [19]. Administration of BAT-derived exosomes to diet-induced obese mice reduces body weight, improves glycemia, and attenuates hepatic lipid deposition [20]. Functional studies revealed diverse roles for these miRNAs. For instance, miR-99b modulates the production of hepatic fibroblast growth factor 21 (FGF21) [19], while cold-induced miR-378a enhances hepatic gluconeogenesis [21]. To identify novel BAT-derived exosomal miRNAs, we purified and sequenced exosomal miRNAs from cultured BAT of wild-type (WT) and obese (*ob/ob)* mice, expanding the known spectrum of metabolically active BAT exosomal miRNAs.

miR-206-3p, a muscle-enriched microRNA (myomiR), regulates myogenic differentiation and regeneration in skeletal muscle [22, 23]. Intriguingly, it is expressed in human BAT and brown adipocytes but is absent in white adipocytes [24]. Unlike myogenic mRNAs that decline during differentiation, miR-206-3p persists throughout brown adipocyte development [25–27]. This persistent expression pattern supports the hypothesis of a shared developmental origin between brown adipocytes and myocytes. Furthermore, although cold acclimation has minimal effects on miR-206-3p expression in BAT [28], its overexpression targets neurotrophins (vascular endothelial growth factor A (VEGFA), brain-derived neurotrophic factor (BDNF), and nerve growth factor (NGF)), leading to reduced core body temperature after cold exposure [29]. Despite existing evidence supporting its role as a BAT marker, the specific functional mechanisms of miR-206-3p in BAT remain unclear. Our sequencing of BAT-derived exosomal miRNAs revealed significantly decreased miR-206-3p levels in *ob/ob* mice compared to WT, prompting us to further investigate the role of miR-206-3p in BAT.

While miR-206-3p has been primarily characterized in muscle, emerging evidence demonstrates its regulatory role in hepatic metabolism, where it suppresses *de novo* lipogenesis, cholesterol biosynthesis, and very-low-density lipoprotein (VLDL) assembly [30, 31]. Despite its potent regulatory effects, endogenous expression of miR-206-3p in hepatocytes is remarkably low, and its cellular source remains unidentified. Given that BAT-derived exosomal miRNAs ameliorate hepatic metabolic dysregulation, we investigate whether BAT-secreted exosomal miR-206-3p mediates BAT–liver crosstalk, potentially revealing novel therapeutic targets for obesity.

## Results

### BAT is a major source of exosomes, and the exosomal miRNA profile shows a decrease in obese mice

**Figure 1:**
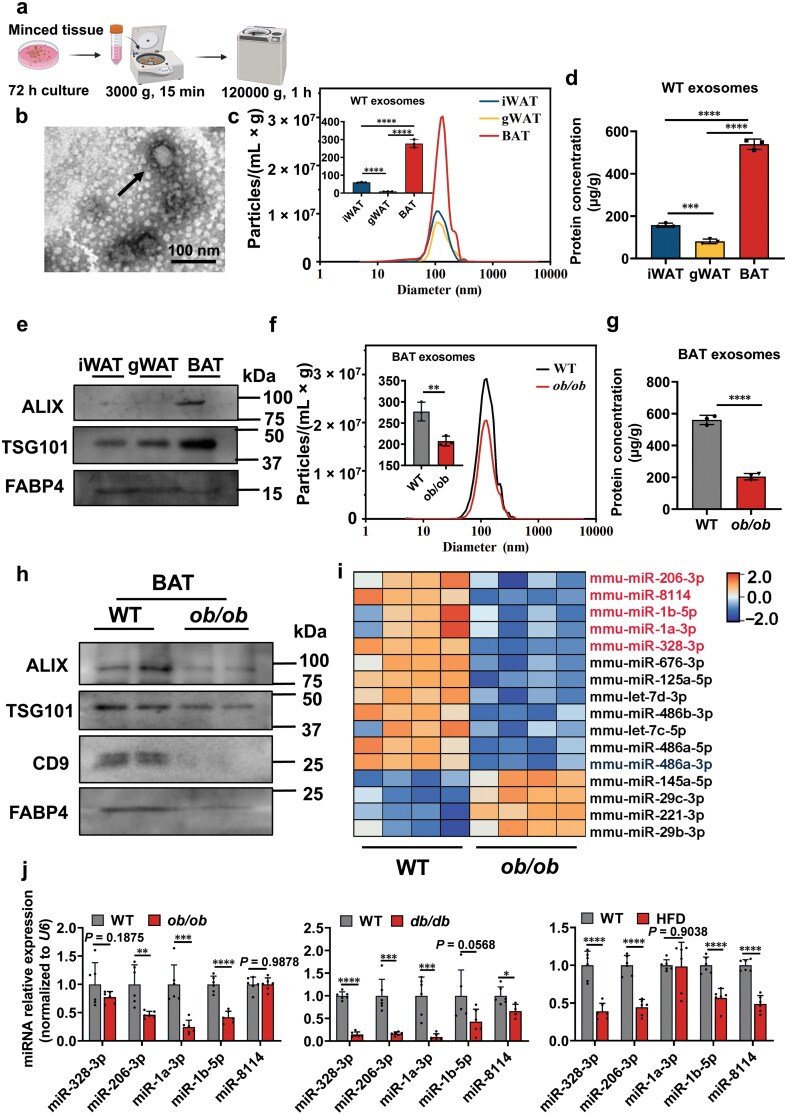
Characterization and the miRNA profile of BAT-derived exosomes from WT and obese mice. (a) Schematic diagram of exosome isolation from adipose tissue. (b) Representative electron micrographs of exosomes isolated from BAT. Scale bar, 100 nm. (c) NTA of exosome size distribution and particle concentration (*n* = 3). (d) Bicinchoninic acid (BCA) analysis of exosome protein concentration (*n* = 3). (e) Western blot detection of exosomal markers ALIX, TSG101, and the fatty acid-binding protein (FABP4) (iWAT mass: 0.34 g/mouse; gWAT mass: 0.39 g/mouse; BAT mass: 0.11 g/mouse). Exosomes were isolated from equal volumes of culture medium derived from iWAT, gWAT, and BAT of 8-week-old male C57BL/6J mice (c−e). (f) NTA of exosome size distribution and particle concentration (*n* = 3). (g) BCA analysis of exosome protein concentration (*n* = 3). (h) Western blot detection of exosomal markers ALIX, TSG101, CD9, and FABP4 (WT BAT mass: 0.12 g/mouse; *ob/ob* BAT mass: 0.38 g/mouse). Exosomes were isolated from equal volumes of BAT culture medium from 8-week-old WT and *ob/ob* mice (f−h). (i) Heatmap of the miRNA sequencing comparing BAT-derived exosomes from WT and *ob/ob* mice (*n* = 4). (j) RT-qPCR validation of candidate miRNAs from miRNA sequencing in *ob/ob*, *db/db*, and HFD-fed mice (*n* = 6). Values are means ± SD. ^*^P < 0.05; ^**^P < 0.01; ^***^P < 0.001 by Student’s *t* test or ANOVA test.

Although adipose tissue is a major source of circulating exosomal miRNAs [19, 32], its secretory capacity remains poorly characterized. To investigate this, we cultured inguinal white adipose tissue (iWAT), gonadal white adipose tissue (gWAT), and BAT from C57BL/6 J male mice for 72 h. Exosomes were isolated from culture supernatants by ultracentrifugation (Fig. 1a) and characterized using transmission electron microscopy (TEM; Fig. 1b) and nanoparticle tracking analysis (NTA; Fig. 1c). BAT secreted significantly more exosomes per gram of tissue than iWAT or gWAT, as evidenced by elevated particle counts (Fig. 1c), protein content (Fig. 1d), and exosomal markers (ALG-2 (apoptosis-linked gene 2)-interacting protein X (ALIX) and tumor susceptibility gene 101 (TSG101); Fig. 1e). Given this robust secretory capacity of BAT, we examined the impact of obesity on exosome production. BAT-derived exosomes from *ob/ob* mice showed reduced particle numbers (Fig. 1f), protein content (Fig. 1g), and exosomal markers (ALIX, TSG101, and cluster of differentiation 9 (CD9); Fig. 1h) compared to that from WT mice, indicating impaired exosomal secretion in obesity.

Given the critical role of miRNAs in post-transcriptional gene regulation within exosomal cargo [33], we performed miRNA sequencing on BAT-derived exosomes from WT and *ob/ob* mice (Fig. 1i). Among 170 differentially expressed miRNAs, 109 were downregulated while only 71 were upregulated in obesity. To identify potential therapeutic candidates, we selected miRNAs meeting two criteria: (i) high basal expression in BAT (Table 1) and (ii) significant downregulation in obesity, as we hypothesized that replenishing these miRNAs could mitigate obesity. Five miRNAs (miR-206-3p, miR-8114, miR-1b-5p, miR-1a-3p, and miR-328-3p) were identified as strong candidates, exhibiting consistent downregulation across three distinct obesity models: *ob/ob* mice, *db/db* mice, and high-fat diet (HFD)-fed mice. These findings were validated using quantitative reverse transcriptase-polymerase chain reaction (RT-qPCR) (Fig. 1j).

**Table 1.** BAT-derived exosomal miRNA profiling.

Gene	Average expression	log_2_(FC)	*P* value
mmu-miR-125a-5p	14.42072	−1.30717	0.000866
mmu-miR-29c-3p	13.33468	1.354493	0.00163
mmu-miR-328-3p	12.19889	−1.80967	5.85E−05
mmu-miR-145a-5p	12.16551	1.146159	0.00299
mmu-let-7d-3p	11.43527	−1.17208	0.002555
mmu-miR-486a-5p	11.0136	−1.05253	0.003929
mmu-miR-486a-3p	11.00383	−1.01195	0.003738
mmu-miR-676-3p	10.98972	−1.52002	0.001106
mmu-miR-486b-3p	10.97953	−1.07782	0.003735
mmu-miR-221-3p	10.94211	1.41479	0.000797
mmu-miR-206-3p	10.66291	−3.22066	0.002035
mmu-miR-29b-3p	10.57574	1.562109	0.003998
mmu-miR-142a-5p	10.11364	2.139098	0.001376
mmu-miR-100-5p	9.634595	1.503787	0.000393
mmu-miR-199a-5p	9.630565	1.696461	0.001821
mmu-miR-193b-5p	9.481162	−1.40666	0.001109
mmu-miR-345-3p	9.17901	−1.07939	0.004256
mmu-miR-199b-5p	9.151225	2.137452	0.007635
mmu-miR-192-5p	8.90878	1.757576	0.000415
mmu-miR-122-5p	8.847472	1.454581	0.001441
mmu-miR-122b-3p	8.822625	1.389215	0.002703
mmu-miR-451a	8.681123	1.066506	0.007515
mmu-miR-335-5p	8.66668	1.876514	0.002046
mmu-miR-1199-3p	8.255567	1.788244	0.00867
mmu-miR-143-5p	8.129181	1.691411	0.004048
mmu-miR-455-5p	8.027948	1.810683	0.000203
mmu-miR-143-3p	7.979873	1.114681	0.007427
mmu-miR-8114	7.859033	−2.61306	2.12E−05
mmu-miR-351-5p	7.826992	−1.1826	0.003243
mmu-miR-203-5p	7.617479	−1.73321	0.001427
mmu-miR-708-3p	7.439377	−1.19436	0.008449
mmu-miR-320-5p	7.344298	1.586969	0.002275
mmu-miR-130b-3p	7.3152	2.18012	0.000444
mmu-miR-1306-5p	6.955261	−1.11598	0.004564
mmu-miR-5121	6.906214	1.587744	0.001074
mmu-miR-3960	6.811343	−1.38211	0.003269
mmu-miR-203b-3p	6.803997	−1.89341	0.002082
mmu-miR-1903	6.774191	1.685978	0.000338
mmu-miR-5099	6.767169	2.433295	0.000155
mmu-miR-1981-5p	6.496153	−1.4335	0.000887
mmu-miR-218-5p	6.476728	1.25379	0.003606
mmu-miR-1943-5p	6.303072	−1.19304	0.002305
mmu-miR-21a-3p	6.215349	2.682834	0.000282
mmu-miR-144-3p	6.112285	1.556686	0.002864
mmu-miR-6238	6.019979	1.087006	0.005339
mmu-miR-503-5p	5.913486	1.398059	0.000481
mmu-miR-1941-5p	5.786185	−2.08793	7.20E−05
mmu-miR-324-5p	5.612622	1.06414	0.007268
mmu-miR-376a-3p	5.531756	2.011291	0.002153
mmu-miR-483-3p	5.491357	−1.92935	0.000873
mmu-miR-17-5p	5.40489	1.325741	0.003807
mmu-miR-6538	5.353528	−2.12793	0.000517
mmu-miR-452-5p	5.288403	1.94264	0.004843
mmu-miR-335-3p	5.288186	1.98876	0.000313
mmu-miR-485-5p	5.268644	−1.44873	0.004938
mmu-miR-129-5p	5.180824	−2.14526	0.001044
mmu-miR-204-5p	5.111432	−1.91402	0.001268
mmu-miR-3076-3p	5.091284	−2.07837	0.000506
mmu-miR-376c-3p	5.066163	1.542827	0.002441
mmu-miR-190b-5p	4.923013	−2.03298	0.008279
mmu-miR-466f-3p	4.499974	−1.34826	0.002381
mmu-miR-30c-2-3p	4.257531	−1.31397	0.007306
mmu-miR-331-3p	4.120141	1.786542	0.004037
mmu-miR-700-3p	4.030677	1.391994	0.004596
mmu-miR-129b-3p	3.99787	−2.23674	0.000613
mmu-miR-3076-5p	3.958509	−2.72078	4.66E−05
mmu-miR-9-3p	3.931011	2.116659	0.003306
mmu-miR-7020-3p	3.756307	2.220696	0.004795
mmu-miR-487b-3p	3.486419	1.441898	0.008559
mmu-miR-8095	3.359144	1.489135	0.007146
mmu-miR-31-3p	3.236204	2.791686	0.001661
mmu-miR-504-5p	3.161023	2.007585	0.003059
mmu-miR-147-3p	2.980156	1.853371	0.000635
mmu-miR-450b-5p	2.86649	1.233543	0.007713
mmu-miR-5130	2.612727	−1.59797	0.004087
mmu-miR-8091	2.252675	−2.03883	0.007809
mmu-miR-7015-3p	2.169173	−1.87182	0.002686
mmu-miR-129-2-3p	1.691744	−2.04848	0.004131

BAT-derived exosomal miRNAs have been ranked in order of their abundance.

### Obesity suppresses miR-206-3p expression and secretion in BAT

**Figure 2:**
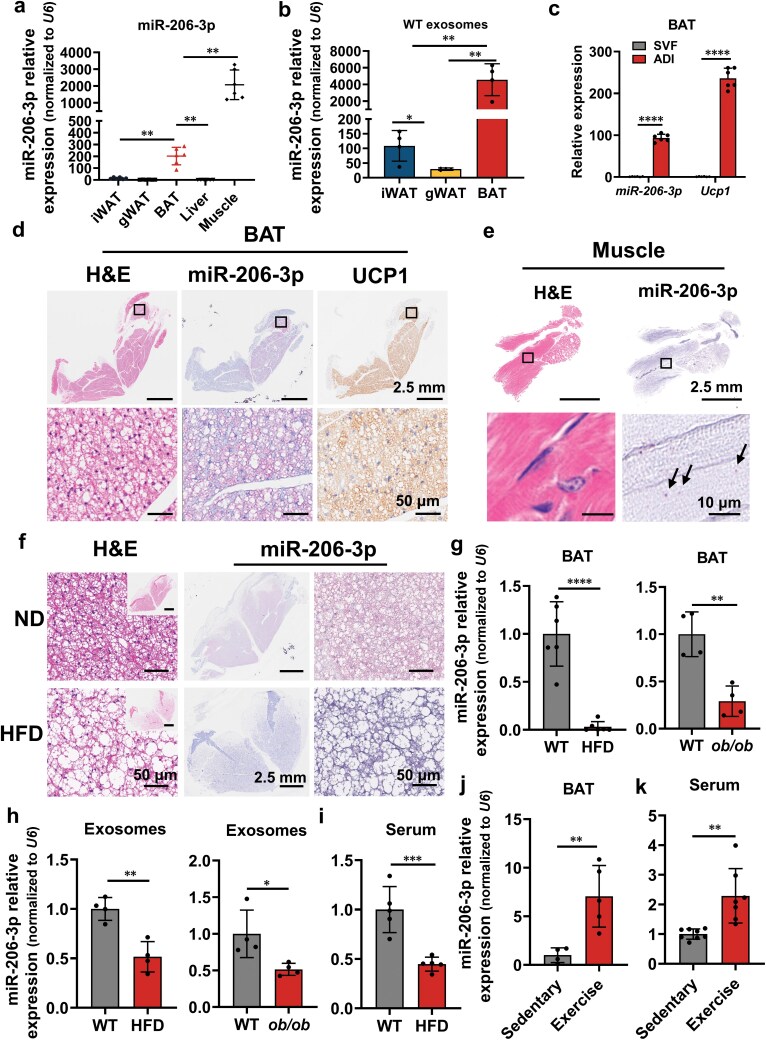
BAT-derived miR-206-3p is downregulated by obesity and upregulated by exercise. (a) RT-qPCR analysis of relative miR-206-3p levels in iWAT, gWAT, BAT, liver, and muscle from male C57BL/6J mice at 8 weeks of age (*n* = 6). (b) FPKM value of miR-206-3p levels in iWAT, gWAT, and BAT-derived exosomal miRNA sequencing from male C57BL/6J mice at 8 weeks of age (*n* = 4). (c) RT-qPCR analysis of the relative levels of miR-206-3p and *Ucp1* in SVF and mature adipocytes (ADI) obtained from BAT of male C57BL/6J mice at 8 weeks of age (*n* = 6). (d) Representative images of H&E staining and miRNAscope staining for miR-206-3p, and immunohistochemical staining for UCP1 in BAT from male C57BL/6J mice at 8 weeks of age. Scale bars, 2.5 mm (up) and 50 μm (BAT). (e) Representative images of H&E staining and miRNAscope staining for miR-206-3p in muscle obtained from male C57BL/6J mice at 8 weeks of age. Scale bars, 2.5 mm (up) and 10 μm (Muscle). (f) Representative images of H&E staining and miRNAscope staining for miR-206-3p in BAT obtained from male C57BL/6J mice fed ND or HFD for 16 weeks starting at the age of 8 weeks. Scale bars, 50 μm (partial) and 2.5 mm (whole). (g−i) RT-qPCR analysis performed to measure the relative levels of miR-206-3p in male C57BL/6J mice fed ND or HFD for 16 weeks starting at the age of 8 weeks, and in *ob/ob* mice at 8 weeks of age. (g) RT-qPCR analysis of relative miR-206-3p levels in BAT from WT and HFD mice (*n* = 6) or WT and *ob/ob* mice (*n *= 4). (h) RT-qPCR analysis of relative miR-206-3p levels in BAT-derived exosomes from WT and HFD mice (*n* = 4) or WT and *ob/ob* mice (*n *= 4). (i) RT-qPCR analysis of relative miR-206-3p levels in serum exosomes from WT and HFD mice (*n* = 4). (j and k) RT-qPCR analysis performed to measure the relative levels of miR-206-3p in male C57BL/6J mice after 28 days of exercise or sedentary lifestyle starting at 8 weeks of age. (j) RT-qPCR analysis of relative miR-206-3p levels in BAT from exercised and sedentary mice (*n* = 5−6). (k) RT-qPCR analysis of relative miR-206-3p levels in serum-derived exosomes from exercised and sedentary mice (*n* = 8). Values are means ± SD. ^*^P < 0.05; ^**^P < 0.01; ^***^P < 0.001 by Student’s *t* test or ANOVA test.

Among the five candidate miRNAs, miR-206-3p and miR-328-3p exhibited the highest basal expression in BAT (Supplementary Fig. S1a). Tissue distribution profiling revealed predominant enrichment of miR-328-3p (Supplementary Fig. S1b) and miR-206-3p (Fig. 2a) in BAT and muscle, with miR-206-3p showing particularly high tissue specificity. Based on these findings, miR-206-3p was selected for further investigation.

Adipose tissue miRNA sequencing indicated that the levels of exosomal miR-206-3p derived from BAT were significantly higher than those in iWAT and gWAT (Fig. 2b). Within BAT, miR-206-3p was preferentially enriched in mature adipocytes compared to the stromal vascular fraction (SVF; Fig. 2c). Using miRNAscope staining, we observed that miR-206-3p signals (red probe) in BAT were predominantly localized to the right region. Higher-magnification analysis revealed a clustered distribution of miR-206-3p within BAT (Fig. 2d), indicating spatial heterogeneity in its expression. In contrast, muscle displayed a uniform punctate distribution pattern (Fig. 2e).

miRNA sequencing revealed significant downregulation of BAT-derived exosomal miR-206-3p in obese mice. Correspondingly, both miRNAscope (Fig. 2f) and RT-qPCR (Fig. 2g) analyses demonstrated reduced miR-206-3p expression within BAT during obesity, paralleling decreased packaging into BAT-derived exosomes (Fig. 2h) and reduced serum exosomal miR-206-3p levels (Fig. 2i). Conversely, miR-206-3p expression in muscle remained unaltered (Supplementary Fig. S2a).

As exercise is a well-established intervention for obesity alleviation, and prior studies report increased serum exosomal miR-206-3p post-exercise (Table 2; Supplementary Fig. S2b) [34], we investigated the tissue origin of circulating exosomal miR-206-3p. Following 28 days of exercise, miR-206-3p expression was specifically upregulated 10-fold in BAT (Fig. 2j) but unchanged in muscle (Supplementary Fig. S2c). This BAT-specific induction was correlated with elevated serum exosomal miR-206-3p (Fig. 2k), indicating BAT as the major source of exercise-induced circulating exosomal miR-206-3p. Collectively, these data suggest that BAT-derived exosomal miR-206-3p plays a critical role in alleviating obesity.

**Table 2.** Changed miRNAs in BAT-derived exosomes  (BDE) and serum-derived exosomes (SDE).

miRNA	Obesity		Exercise	
	BDE		SDE	
	Fold change (obese versus WT)	*P* value	Fold-change (exercise versus sedentary)	*P* value
mmu-miR-30d-5p	−0.845	0.018	2.24	0.035
mmu-miR-133a-3p	*−*0.0995	0.797	9.46	0.006
**mmu-miR-206-3p**	**−3.221**	**0.002**	**9.56**	**0.006**
mmu-let-7g-5p	*−*0.048	0.9074	*−*7.27	0.011
mmu-miR-192-5p	1.758	0.000415	*−*2.79	0.0067
mmu-miR-320-5p	1.587	0.002	*−*2.25	0.108

Comparative analyses of miRNAs in obese mice relative to lean mice from BAT-derived exosomes (Obese versus WT) and exercise mice relative to sedentary mice from serum-derived exosomes are shown.

### BAT-derived exosomal miR-206-3p is delivered to the liver

**Figure 3:**
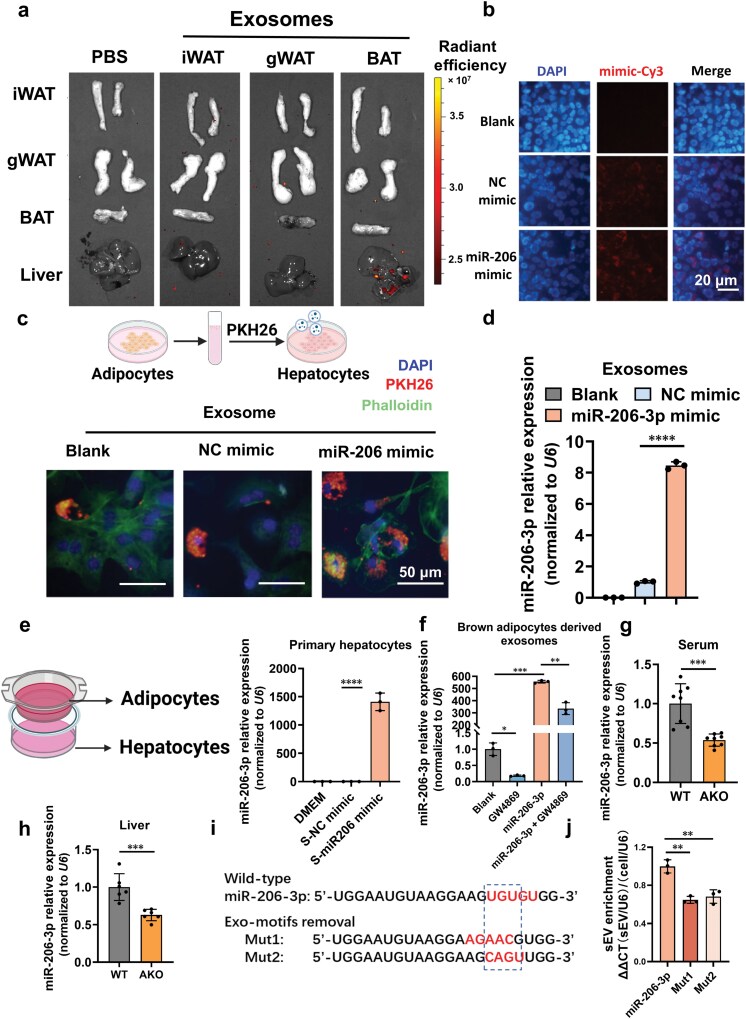
BAT exosome-mediated hepatic delivery of miR-206-3p. (a) C57BL/6J mice injected with PBS or 50 μg PKH26-labeled exosomes derived from iWAT, gWAT, or BAT and subjected to IVIS analysis at 24 h after intravenous (i.v.) injection. (b) Representative images of primary hepatocytes transfected with Cy3-labeled NC mimic or miR-206-3p mimic for 24 h. Scale bar, 20 μm. (c) Representative images of primary hepatocytes co-cultured with brown adipocytes-derived miR-206-3p-overexpressing exosomes. Brown adipocytes were transfected with miR-206-3p mimic or NC mimic. The exosomes secreted by adipocytes were labeled with PKH26 and co-cultured with primary hepatocytes for 24 h. Scale bar, 50 μm. (d) RT-qPCR analysis of relative miR-206-3p levels in brown adipocytes derived exosomes. Primary brown adipocytes were transfected with miR-206-3p mimic or NC mimic for 24 h, followed by induction of cell differentiation and collection of culture supernatants for exosome isolation (*n* = 3). (e) RT-qPCR analysis of relative miR-206-3p levels in primary hepatocytes pretreated with the conditioned media from mature adipocytes transfected with NC mimic or miR-206-3p mimic. Mature adipocytes were seeded in the Transwell inserts, while primary hepatocytes were seeded in the bottom chamber (*n* = 3). (f) RT-qPCR analysis of relative miR-206-3p levels in exosomes derived from brown adipocytes treated with GW4869 (10 μmol/L) and miR-206-3p mimic (*n* = 3). During the adipocyte differentiation process, the exosome biogenesis inhibitor GW4869 was continuously added. (g) RT-qPCR analysis of relative miR-206-3p levels in serum-derived exosomes from WT (miR-206^flox/flox^) and AKO (miR-206^flox/flox^; Adipoq-Cre) mice at 8 weeks of age (*n* = 8). (h) RT-qPCR analysis of relative miR-206-3p levels in the liver of WT and AKO mice (*n* = 8). (i) Table depicting the sequence of the wild-type miR-206-3p and the mutated sequence without exo-motif UGUGU. Red text in the sequence indicates changed nucleotides in the guide strand of the miRNA used to remove the exo-motif UGUGU. Nucleotides in the passenger strand were also modified to maintain miRNA structure. (j) The small extracellular vesicle (sEV) enrichment calculated as the ratio of sEV expression divided by cellular expression for each of the constructs expressed in and secreted from brown adipocytes. The miRNA expression was normalized to the expression of *U6* (*n *= 3). Values are means ± SD. ^*^P < 0.05; ^**^P < 0.01; ^***^P < 0.001; ^****^P < 0.0001 by Student’s *t* test or ANOVA test.

To delineate the *in vivo* distribution of the BAT-derived exosomal miR-206-3p, exosomes isolated from iWAT, gWAT, and BAT were labeled with PKH26 and intravenously injected into mice (Fig. 3a). Hepatic accumulation of BAT-derived exosomes was significantly greater than those from iWAT or gWAT. We further confirmed *in vitro* that primary hepatocytes internalized both Cy3-labeled miR-206-3p mimics and exosomes containing this mimic (Fig. 3b and c). To investigate the exosome-dependent delivery mechanism of miR-206-3p, we harvested exosomes from primary brown adipocytes transfected with miR-206-3p mimic or negative control (NC) mimic. These exosomes contained significantly elevated miR-206-3p levels relative to the controls (Fig. 3d). Furthermore, treatment of primary hepatocytes with conditioned media from miR-206-3p-overexpressing adipocytes increased intracellular miR-206-3p levels (Fig. 3e). Importantly, pharmacological inhibition of exosome biogenesis using GW4869 in adipocytes significantly reduced miR-206-3p levels in secreted exosomes (Fig. 3f). Critically, adipose tissue-specific miR-206-3p knockout (AKO) mice displayed significantly diminished miR-206-3p levels in both serum and liver tissue relative to WT controls (Fig. 3g and h). Taken together, these findings confirm that BAT-derived miR-206-3p is secreted via exosomes and subsequently internalized by hepatocytes.

To elucidate the mechanism governing miR-206-3p secretion into exosomes, we investigated its specific sorting mechanism. Previous studies have demonstrated that miRNAs containing specific exo-motifs are selectively packaged into exosomes, with tissue-specific variations in these motifs [35, 36]. Notably, the miR-206-3p sequence contains “UGUGU”, which corresponds to a predicted BAT-specific exo-motif [35]. To functionally validate this motif, we introduced mutations (Mut1 and Mut2) into the UGUGU exo-motif (Fig. 3i) and evaluated the abundance of the mutant miR-206-3p in exosomes (Fig. 3j). Disruption of the UGUGU motif markedly reduced Mut1 and Mut2 enrichment within secreted exosomes, indicating that the intact UGUGU motif is critical for the selective sorting of miR-206-3p into exosomes in brown adipocytes.

### miR-206-3p deficiency exacerbates metabolic disorder

To clarify the role of miR-206-3p *in vivo*, we generated AKO mice by crossing miR-206^flox/flox^ mice with Adipoq-Cre mice (Supplementary Fig. S3a). AKO mice exhibited significantly reduced miR-206-3p expression in iWAT, gWAT, and BAT compared to WT controls (Supplementary Fig. S3b). When maintained on normal chow diet (ND), WT and AKO mice showed comparable body weight, adipose tissue mass, and liver weight (Supplementary Fig. S3c−e), with no discernible differences in the morphology of iWAT, gWAT, or BAT (Supplementary Fig. S3d). Importantly, miR-206 deficiency did not affect glucose tolerance, insulin sensitivity, or hepatic triglyceride (TG) content under basal dietary conditions (Supplementary Fig. S3f−h).

**Figure 4:**
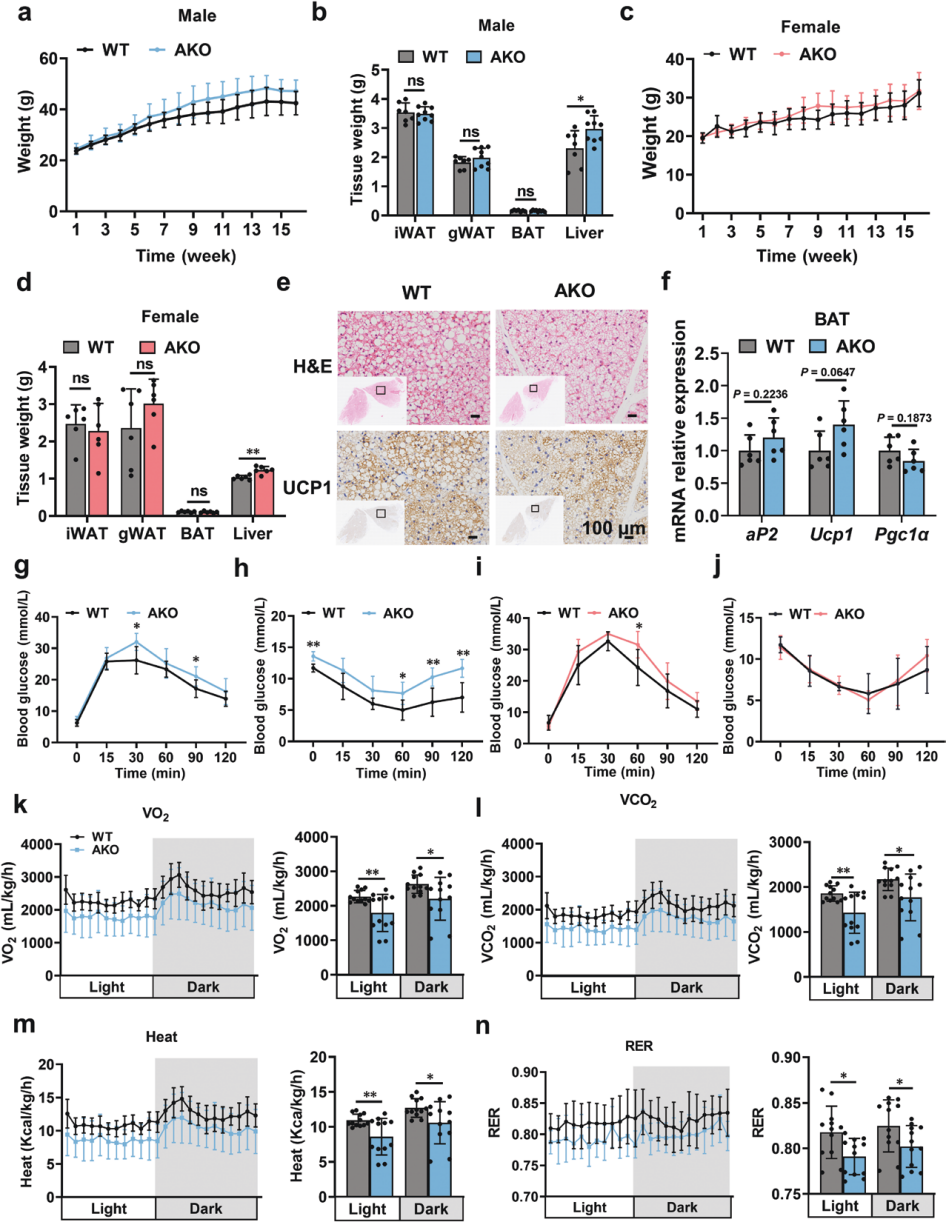
Adipose-specific miR-206 deletion exacerbates obesity-associated metabolic dysfunction in mice. (a) Body weight of WT and AKO male mice after HFD (*n* = 8). (b) The iWAT, gWAT, BAT, and liver weight in the indicated male mice (*n *= 7−9). (c) Body weight of WT and AKO female mice after HFD (*n *= 8). (d) The iWAT, gWAT, BAT, and liver weight in the indicated female mice (*n *= 8). (e) Representative images of H&E and UCP1 staining in BAT from WT and AKO male mice. Scale bars, 100 μm. (f) Relative mRNA expression of adipogenesis (*aP2*) and thermogenic (*Ucp1* and *Pgc1α*) genes in BAT (*n *= 6). (g) Glucose concentrations during the GTT in male mice after 11 weeks of HFD feeding (*n *= 6). (h) Glucose concentrations during the ITT in male mice after 10 weeks of HFD feeding (*n *= 6). (i) Glucose concentrations during the GTT in female mice after 11 weeks of HFD feeding (*n* = 6). (j) Glucose concentrations during the ITT in female mice after 10 weeks of HFD feeding (*n* = 6). (k) Oxygen consumption rate (VO_2_) measured by metabolic cages for male mice after 16 weeks of HFD feeding (*n *= 12). (l) Carbon dioxide consumption rate (VCO_2_) measured by metabolic cages for male mice after 16 weeks of HFD feeding (*n *= 12). (m) Heat production measured by metabolic cages for male mice after 16 weeks of HFD feeding (*n *= 12). (n) Respiratory exchange ratio (RER) measured by metabolic cages for male mice after 16 weeks of HFD feeding (*n *= 12). The 8-week-old WT and AKO mice were fed HFD for 16 weeks before being sacrificed for analysis. Values are means ± SD. ns, no significance; ^*^P < 0.05; ^**^P < 0.01; ^***^P < 0.001; ^****^P < 0.0001 by Student’s *t* test or ANOVA test.

To define the role of miR-206-3p in obesity, 8-week-old WT and AKO mice were fed HFD for 16 weeks. Male AKO mice showed no significant differences in body weight gain or adipose tissue mass compared to WT mice (Fig. 4a and b), but exhibited significantly increased liver weight (Fig. 4b). Female AKO mice exhibited a similar phenotype (Fig. 4c and d). Given sustained miR-206-3p expression during brown adipocyte differentiation [26], we performed CRISPR-Cas9-mediated knockdown in pre-brown adipocytes (Supplementary Fig. S4a−c). This genetic perturbation did not affect adipogenic differentiation or browning process. Consistent with these *in vitro* findings, local injection of adenovirus overexpressing miR-206 into BAT *in vivo* failed to modulate lipid droplet content or thermogenic gene expression (Supplementary Fig. S4d and e). Corroborating these findings, genetic ablation of miR-206-3p did not impair BAT function (Fig. 4e and f). Collectively, these findings suggest that although miR-206-3p is abundantly expressed in brown adipocytes, it is dispensable for adipocyte differentiation and thermogenic programming but may serve as a potential biomarker.

We then analyzed WT and AKO mice after 16 weeks of HFD feeding. Male AKO mice showed impaired glucose tolerance (Fig. 4g) and insulin resistance (Fig. 4h), but these phenotypes were not observed in female AKO mice (Fig. 4i and j). Additionally, AKO mice showed decreased oxygen consumption (Fig. 4k), carbon dioxide release (Fig. 4l), heat production (Fig. 4m), and respiratory exchange ratio (Fig. 4n). Collectively, these data demonstrate that adipose-specific miR-206-3p ablation exacerbates metabolic dysregulation, including glucose intolerance, insulin resistance, and suppressed energy expenditure in response to HFD challenge.

### Adipose-specific miR-206-3p deficiency exacerbates HFD-induced hepatic steatosis

**Figure 5:**
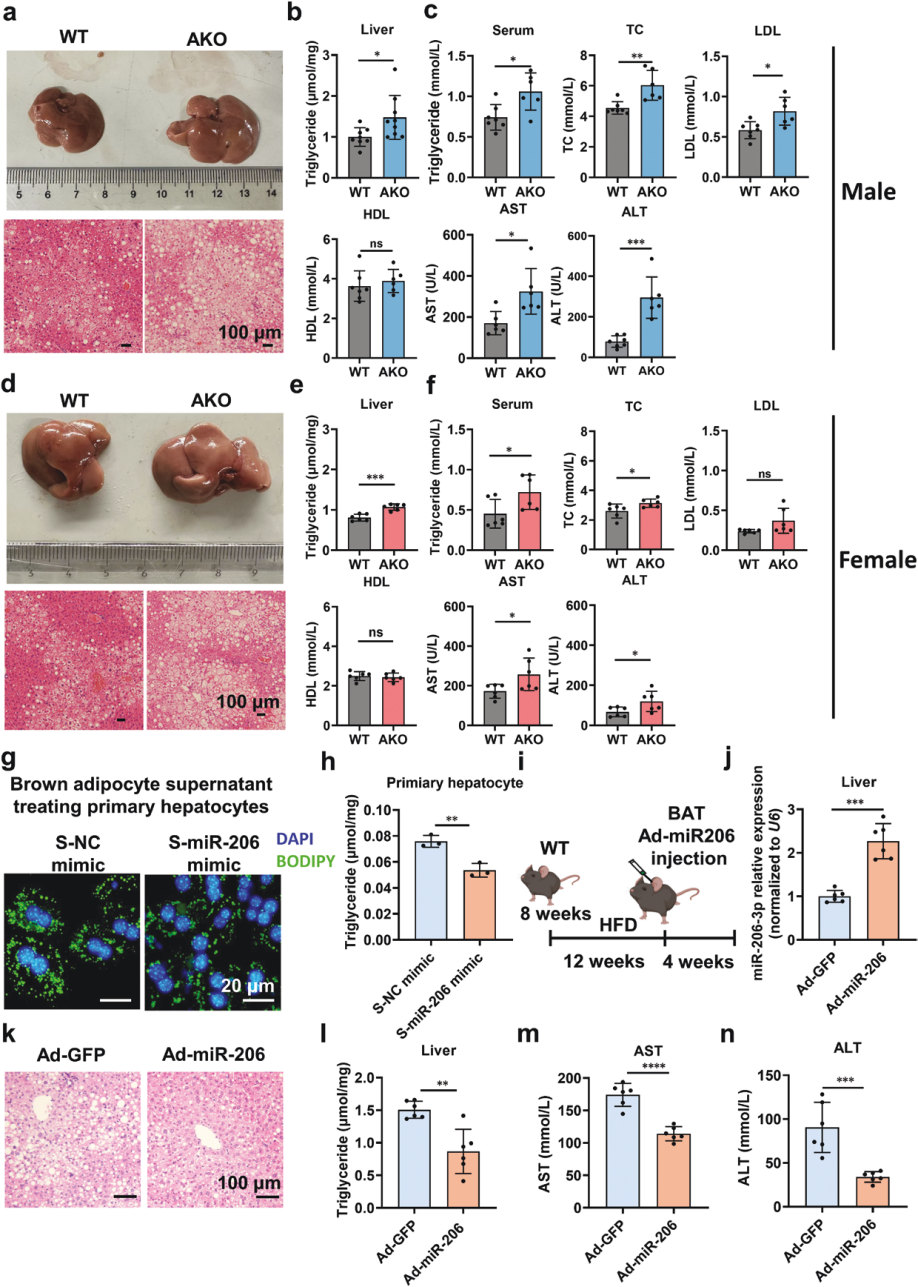
miR-206-3p deficiency exacerbates hepatic steatosis. (a) Gross morphology of livers from 24-week-old WT and AKO male mice fasted for 16 h and H&E staining of liver sections from these mice (*n* = 7−9). Scale bar, 100 μm. (b) Liver TG levels in male mice (*n* = 7−9). (c) The levels of serum TG, TC, LDL, HDL, AST, and ALT in male mice (*n *= 6). (d) Gross morphology of livers from 24-week-old WT and AKO female mice fasted for 16 h and H&E staining of liver sections from these mice (*n *= 7−9). Scale bar, 100 μm. (e) Liver TG levels in female mice (*n *= 6). (f) The levels of serum TG, TC, LDL, HDL, AST, and ALT in female mice (*n *= 6). The 8-week-old WT and AKO mice were fed HFD for 16 weeks before being sacrificed for analysis (a−f). (g) Representative images of BODIPY (4,4-difluoro-4-bora-3a,4a-diaza-s-indacene) and DAPI fluorescence in primary hepatocytes. Scale bar, 20 μm. (h) TG levels in primary hepatocytes (*n *= 3). Brown adipocytes were transfected with miR-206-3p mimic or NC mimic for 24 h. Following differentiation induction, culture supernatants were harvested and co-incubated with primary hepatocytes for 24 h (g and h). (i−n) Impact of miR-206-overexpressing adenovirus injection into the BAT on hepatic lipid accumulation and liver function in HFD-fed mice. (i) Schematic diagram of local injection of miR-206-overexpressing adenovirus into the BAT of HFD-fed mice. (j) RT-qPCR analysis of relative miR-206-3p levels in liver (*n *= 6). (k) Representative H&E staining of liver sections. Scale bar, 100 μm. (l) TG levels in liver (*n *= 6). (m) The levels of serum AST in mice (*n *= 6). (n) The levels of serum ALT in mice (*n *= 6). The 8-week-old male C57BL/6J mice were fed HFD for 16 weeks and injected with Ad-GFP or Ad-miR-206 into BAT starting from week 13, once weekly (i−n). Values are means ± SD. ^*^P < 0.05; ^**^P < 0.01; ^***^P < 0.001; ^****^P < 0.0001 by Student’s *t* test or ANOVA test.

Given the significantly increased liver weights observed in AKO mice compared to WT mice, we performed hematoxylin and eosin (H&E) staining of liver sections from HFD-fed male and female mice (Fig. 5a and d). TG quantification analysis revealed substantial hepatic lipid accumulation in AKO mice (Fig. 5b and e). Serum analysis further demonstrated significantly elevated levels of TG, total cholesterol (TC), low-density lipoprotein (LDL), aspartate aminotransferase (AST), and alanine aminotransferase (ALT) in AKO mice (Fig. 5c and f). These findings indicate that adipose tissue-specific knockout of miR-206 exacerbates hepatic steatosis and associated metabolic dysregulation.

*In vitro*, treatment of primary hepatocytes with conditioned media derived from miR-206-3p-overexpressing adipocytes significantly reduced both cell size and intracellular lipid accumulation compared to control media (Fig. 5g and h). Direct overexpression of miR-206-3p in primary hepatocytes also significantly inhibited lipid accumulation (Supplementary Fig. S5a and b), and this inhibition was abolished by miR-206-3p inhibitors. To investigate this effect *in vivo*, HFD-fed mice received intravenous administration of miR-206-3p-expressing adenovirus for 4 weeks (Supplementary Fig. S5c−e). This intervention markedly attenuated hepatic lipid accumulation (Supplementary Fig. S5f and g), and significantly reduced serum AST and ALT levels (Supplementary Fig. S5h and i), indicating a hepatoprotective role for miR-206-3p in HFD-induced liver dysfunction.

Furthermore, local injection of miR-206-overexpressing adenovirus into the BAT of HFD-fed mice (Fig. 5i) resulted in significant upregulation of miR-206-3p levels in the liver (Fig. 5j). This was accompanied by a marked reduction in hepatic lipid accumulation and TG content (Fig. 5k and l), and decreased serum AST and ALT levels (Fig. 5m and n). Collectively, these findings demonstrate that BAT-derived miR-206-3p plays a protective role in attenuating hepatic lipid accumulation and improving liver function.

### miR-206-3p regulates hepatic lipid accumulation by targeting the pentose phosphate pathway (PPP)

**Figure 6:**
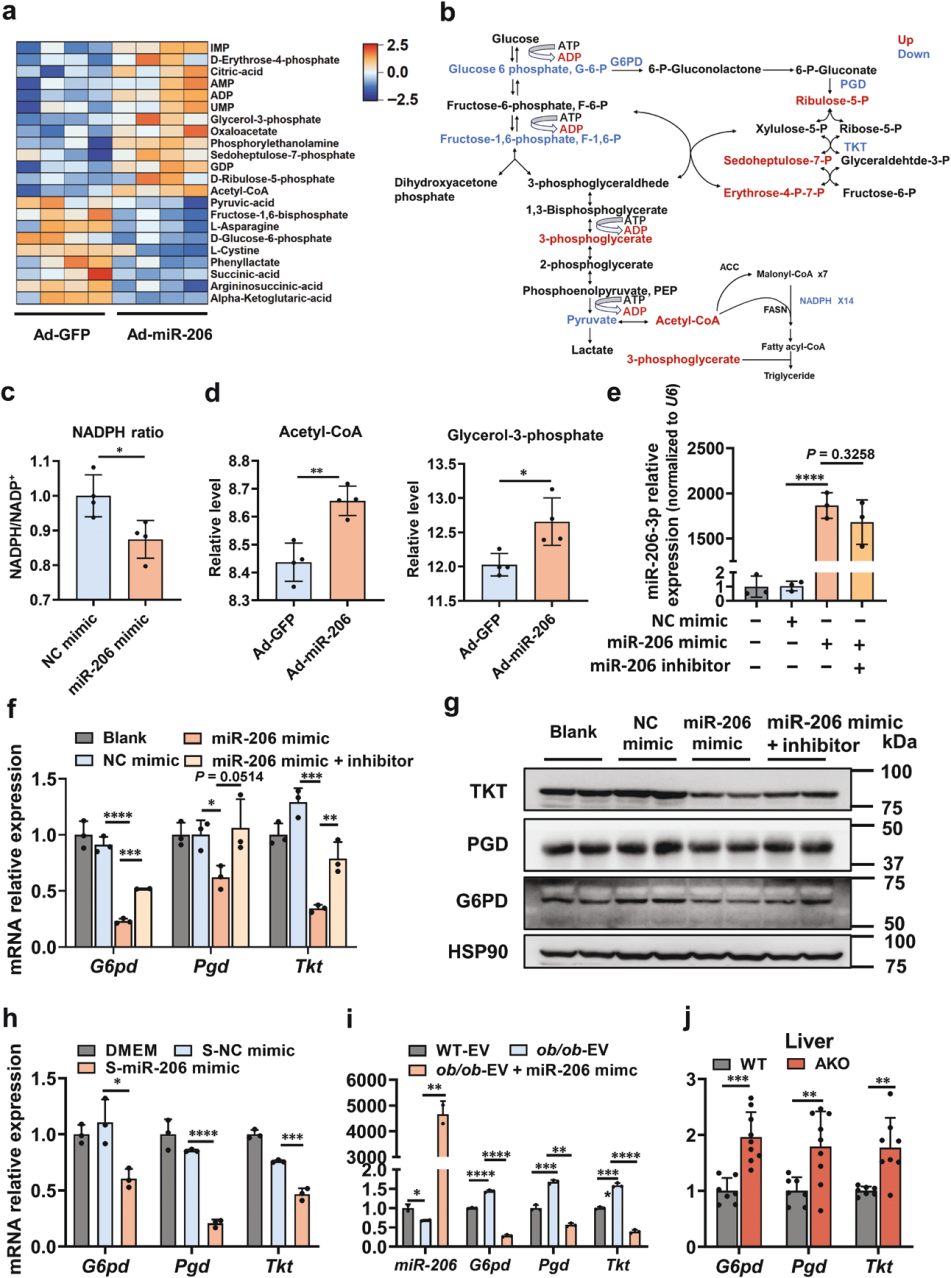
miR-206-3p attenuates lipogenesis by targeting the PPP. (a) Heatmap of liver metabolomics analysis. The 8-week-old male C57BL/6J mice were fed HFD for 16 weeks and injected via tail vein with Ad-GFP or Ad-miR-206 once weekly beginning at week 13 (*n* = 4). (b) Metabolomics identified up-regulated (red) and down-regulated (blue) metabolites in Ad-miR-206-injected mice. (c) NADPH/NADP^+^ ratios in primary hepatocytes transfected with NC mimic or miR-206 mimic (*n* = 3). (d) Relative levels of acetyl-CoA and glycerol-3-phosphate identified by metabolomics (*n* = 4). (e) RT-qPCR analysis of relative miR-206-3p levels in primary hepatocytes (*n* = 3). (f) Relative mRNA expression of PPP genes (*G6pd, Pgd,* and *Tkt*) in primary hepatocytes (*n* = 3). (g) Western blot analysis of PPP proteins (G6PD, PGD, and TKT) in primary hepatocytes. Primary hepatocytes were transfected with NC mimic, miR-206 mimic, or miR-206-3p mimic plus miR-206-3p inhibitor. After 48 h, cells were harvested for RNA and protein analyses (e−g). (h) Relative mRNA expression of PPP genes (*G6pd, Pgd,* and *Tkt*) analyzed in hepatocytes incubated with conditioned medium from mature adipocytes (*n* = 3). Brown adipocytes were transfected with miR-206 mimic or NC mimic on day 3. The culture medium was harvested until day 8 after induction for further studies. (i) Relative mRNA expression of PPP genes (*G6pd, Pgd,* and *Tkt*) in hepatocytes pretreated with BAT-derived exosomes (5 μg) from 8-week-old WT and *ob/ob* mice (*n* = 3). (j) Relative mRNA expression of PPP genes (*G6pd, Pgd,* and *Tkt*) in the livers of the WT and AKO male mice at 8 weeks of age (*n* = 7−9). Values are means ± SD. ^*^P < 0.05; ^**^P < 0.01; ^***^P < 0.001; ^****^P < 0.0001 by Student’s *t* test or ANOVA test.

Previous studies indicate that miR-206-3p targets the protein tyrosine phosphatase non-receptor type 1 (*Ptpn1*) to inhibit hepatic lipid synthesis [31], though this regulation appears indirect. Additionally, miR-206-3p suppresses tumor cell proliferation through PPP targeting [37]. To elucidate its role in fatty liver amelioration, we performed hepatic metabolomic profiling in mice injected with Ad-GFP or Ad-miR-206 via the tail vein. Metabolomic analysis revealed that miR-206-3p overexpression significantly elevated nucleotide-related metabolites (IMP, ADP, AMP, UMP, and ribose-5-phosphate [R-5-P]; Fig. 6a and b) but reduced nicotinamide adenine dinucleotide phosphate (NADPH) levels (Fig. 6c). Notably, reduced NADPH occurred alongside accumulation of lipogenic precursors (acetyl-CoA and glycerol-3-phosphate; Fig. 6d). This observation prompted us to assess the regulatory role of miR-206-3p in *de novo* lipogenesis. RT-qPCR analysis revealed significant downregulation of core lipogenic genes (sterol regulatory element-binding transcription factor 1c (*Srebp1c*), acetyl-CoA carboxylase (*Acc*), and fatty acid synthase (*Fasn*); Supplementary Fig. S6a), whereas no significant changes were observed in β-oxidation-related genes (antioxidant protein 1 (*Atox1*) and carnitine O-palmitoyltransferase 1 (*Cpt1*); Supplementary Fig. S6b).

As the PPP serves as a primary cellular NADPH source, we investigated whether miR-206-3p regulates hepatic metabolism through this pathway. Treatment of hepatocytes with miR-206-3p mimic significantly suppressed PPP activity at both RNA and protein levels; this effect was abolished by miR-206-3p inhibitor co-treatment (Fig. 6e–g). In addition, conditioned medium from miR-206-3p-overexpressing adipocytes inhibited PPP activity in primary hepatocytes (Fig. 6h). To examine BAT-derived exosome-mediated regulation, we treated hepatocytes with exosomes isolated from WT and *ob/ob* mice. Exosomes from *ob/ob* mice significantly reduced miR-206-3p levels and enhanced PPP-related gene expression compared to WT-derived exosomes (Fig. 6i). Consistent with these findings, genetic ablation of miR-206-3p markedly upregulated hepatic PPP activity *in vivo* (Fig. 6j). Similarly, obesity enhanced hepatic PPP activity but induced no significant alterations in BAT or muscle (Supplementary Fig. S7a−d). Using luciferase reporter assays, we confirmed direct binding of miR-206-3p to the 3′-UTRs of glucose-6-phosphate dehydrogenase (*G6pd*) and transketolase (*Tkt*), which suppressed their post-transcriptional expression (Supplementary Fig. S8). These results demonstrate that miR-206-3p downregulates PPP activity by directly suppressing G6PD and TKT expression.

Metabolomic elevation of nucleotide intermediates (IMP, ADP, AMP, UMP, and R-5-P; Fig. 6a and b) prompted transcriptomic analysis in the liver. RNA sequencing (RNA-seq) revealed enrichment of hepatocyte proliferation pathways following miR-206-3p overexpression (Supplementary Fig. S9a), which was corroborated by increased Ki67-positive cell density (Supplementary Fig. S9b). These data suggest that miR-206-3p enhances hepatocyte proliferation potentially through nucleotide precursor availability.

### miR-206-3p plays a role as an MAFLD-related metabolic regulator

**Figure 7:**
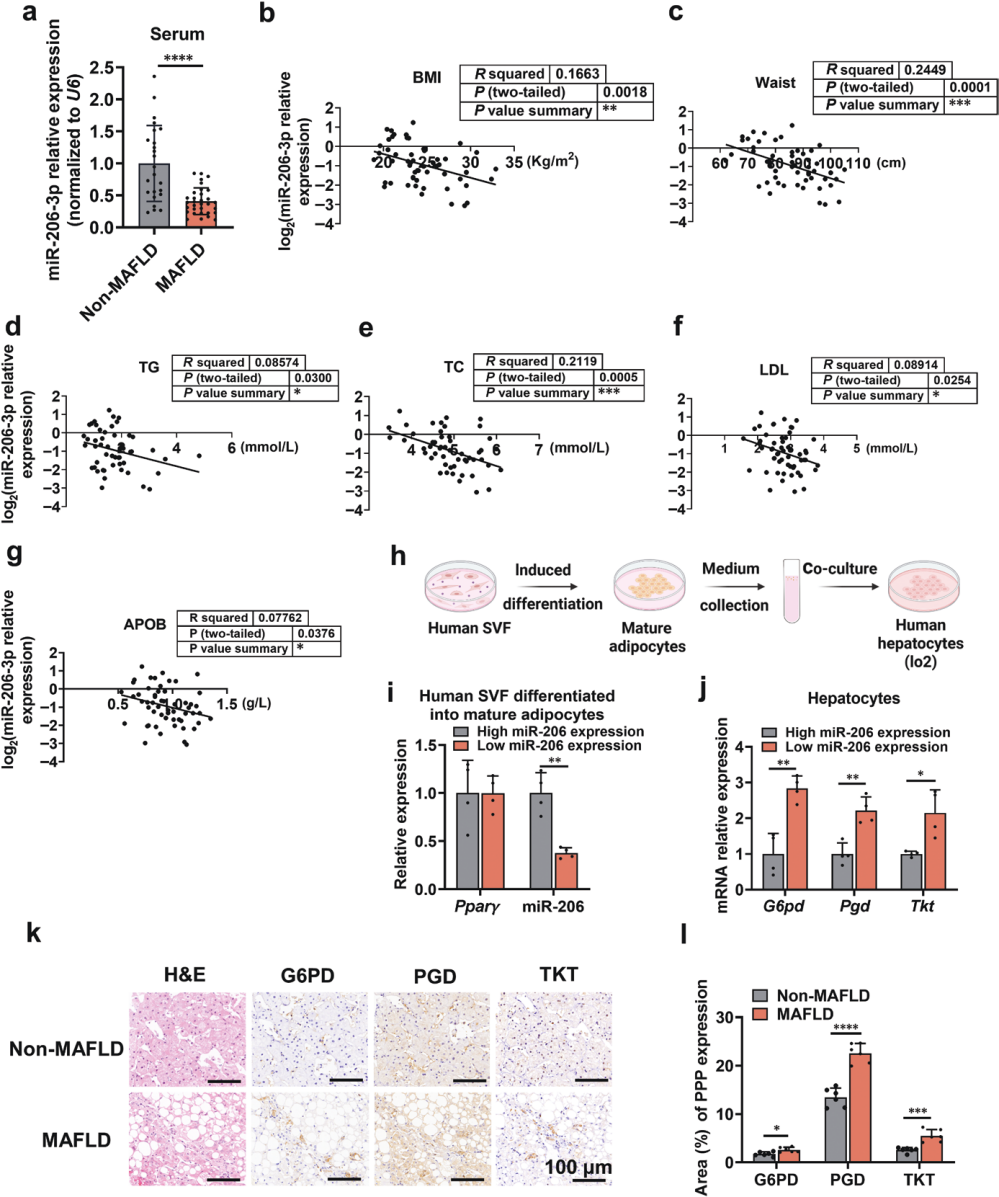
miR-206-3p is reduced in human obesity. (a) qPCR analysis of relative miR-206-3p levels in serum-derived exosomes from healthy controls (Non-MAFLD, *n* = 24) and MAFLD patients (*n* = 32). (b−g) Correlations between (b) BMI, (c) waist, (d) TG, (e) TC, (f) LDL, and (g) APOB and the expression levels of miR-206-3p in serum-derived exosomes (*n *= 50). (h) Flowchart of co-culture between human SVF and hepatocytes. (i) Relative *Pparγ* and miR-206-3p expression in human mature adipocytes (*n *= 4). (j) Relative mRNA expression of PPP genes (*G6pd, Pgd,* and *Tkt*) in hepatocytes incubated with conditioned medium from mature adipocytes (*n *= 4). Human-derived SVF was induced to differentiate into mature adipocytes, and the culture medium was harvested on day 8 after induction for further study (h−j).(k) Representative H&E, G6PD, PGD, and TKT staining in liver sections of MAFLD patients and Non-MAFLD controls. Scale bar, 100 μm. (l) Quantification of positive area for immunohistochemistry staining in the liver (*n *= 6). Values are means ± SD. ^*^P < 0.05; ^**^P < 0.01; ^***^P < 0.001; ^****^P < 0.0001 by Student’s *t* test or ANOVA test.

To evaluate the clinical relevance of miR-206-3p in MAFLD, we analyzed serum samples from MAFLD patients and healthy controls. We found that serum miR-206-3p levels were significantly reduced in MAFLD patients compared to healthy individuals (Fig. 7a) and were inversely correlated with body mass index (BMI), waist circumference (Fig. 7b and c), and key serum lipid markers, including TG, TC, LDL, and apolipoprotein B (APOB) (Fig. 7d−g). In contrast, no significant correlations were observed with high-density lipoprotein (HDL), apolipoprotein A (APOA), or glucose levels (Supplementary Fig. S10a−c).

To establish functional relevance, we differentiated human SVF into mature adipocytes and stratified them based on high or low miR-206-3p expression (Fig. 7h and i). Hepatocytes treated with supernatants from adipocytes with high miR-206-3p expression exhibited significant downregulation of the PPP (Fig. 7j). Immunohistochemical analysis of liver biopsies confirmed marked upregulation of key PPP enzymes in MAFLD patients (Fig. 7k and l). Taken together, these findings establish miR-206-3p as an MAFLD-related metabolic regulator. Reduced miR-206-3p levels correlate with clinical manifestations of obesity and dyslipidemia, and may further promote hepatic lipid accumulation by derepressing the PPP.

## Discussion

BAT was traditionally considered a “low-secretion” organ. However, the discovery of active BAT depots in adult humans over a decade ago prompted a reevaluation of its secretory functions [38]. Emerging research on exosomes has clarified the regulatory roles of BAT-derived exosomal miRNAs. Studies demonstrate that BAT-derived exosomal miR-99b specifically targets hepatic FGF21 to modulate adipose thermogenesis [19], while miR-99a attenuates hepatic oxidative stress by inhibiting NADPH oxidase 4 (NOX4) [39]. Cold stimulation activates BAT and upregulates exosomal miR-132-3p, which suppresses sterol regulatory element binding transcription factor 1 (Srebf1) and lipogenic gene expression [40]. Furthermore, BAT-derived miR-30b alleviates diabetic nephropathy [41], whereas miR-125b-5p, miR-128-3p, and miR-30d-5p confer cardioprotection by inhibiting pro-apoptotic mitogen-activated protein kinases (MAPK) signaling [42]. Collectively, these findings establish BAT-derived exosomes as critical regulators of systemic metabolism.

Given the unique properties of BAT, we screened for exosomal miRNAs mitigating obesity and identified five significantly downregulated candidates: miR-206-3p, miR-328-3p, miR-8114, miR-1a-3p, and miR-1b-5p. Existing studies demonstrate that miR-8114 downregulates renal aquaporin 2 (AQP2) (exacerbating diabetic nephropathy) [43] and is upregulated in senescent pancreatic β-cells [44]; miR-328-3p promotes brown adipocyte differentiation [45]; and the miR-1 family primarily induces cardiomyocyte hypertrophy [46] and apoptosis [47]. We focused on miR-206-3p due to its high expression in BAT and muscle. Both *in vivo* and *in vitro* experiments showed that BAT-specific miR-206-3p overexpression significantly attenuates hepatic lipid accumulation, suggesting therapeutic potential against obesity-associated steatosis.

Our study demonstrates that obesity significantly reduces both miR-206-3p expression in BAT and its exosomal secretion. Given that adipose-specific Dicer knockout impairs miR-206-3p biogenesis [19] and obesity downregulates BAT Dicer expression [45], we propose that Dicer mediates obesity-induced miR-206-3p suppression. Notably, obesity upregulates other BAT miRNAs, indicating complex regulatory mechanisms requiring further study.

To assess miR-206-3p dynamics under physiological stress, we examined its response to cold exposure, a potent BAT activator [48]. Cold exposure downregulated miR-206-3p in iWAT and BAT (data not shown), which is consistent with its role in suppressing thermogenesis through targeting VEGFA and BDNF [29]. Although exercise elevates circulating miR-206-3p [34], its cellular origin remains unclear. We observed that exercise induced transcriptional upregulation of miR-206-3p in BAT but not skeletal muscle, suggesting that BAT-derived exosomal miR-206-3p could contribute to circulating miR-206-3p levels. Collectively, these findings indicate that exercise-induced BAT-specific miR-206-3p production may represent a therapeutic target for alleviating obesity and metabolic syndrome.

Adipose-derived exosomal miRNAs are essential regulators of systemic metabolic homeostasis [32]. To investigate the function of BAT-derived exosomal miR-206-3p in systemic circulation, we note that, while prior studies demonstrate its capacity to alleviate hepatocyte lipid accumulation [30, 31, 49, 50], endogenous miR-206-3p was nearly undetectable in the liver. Given that BAT-derived exosomes can target the liver, we hypothesized that BAT-derived exosomal miR-206-3p traffics to the liver. Intravenous administration of PKH26-labeled BAT-derived exosomes to mice confirmed preferential hepatic accumulation. Crucially, treatment with conditioned media or exosomes from miR-206-3p-overexpressing brown adipocytes elevated miR-206-3p levels in hepatocytes. Longitudinal adenovirus-mediated miR-206-3p overexpression in BAT of HFD-fed mice significantly reduced hepatic steatosis. These findings demonstrate that BAT-derived exosomal miR-206-3p is taken up by the liver and functionally ameliorates lipid accumulation.

Beyond BAT, emerging evidence indicates that exosomes from extrahepatic tissues undergo systemic trafficking to the liver and mediate biologically significant effects. Intravenously administered exosomes exhibit tissue-specific biodistribution and metabolic regulatory functions. Serum-derived exosomes from obese or exercise-trained mice show preferential accumulation in the liver, gWAT, and skeletal muscle [51]. Critically, circulating exosomal miR-133b-3p from exercise-trained mice enhances hepatic insulin sensitivity. In obesity, visceral adipose tissue (VAT) macrophage-derived exosomes traffic to the liver, adipose tissue, and muscle, where exosomal miR-155 exacerbates insulin resistance [52]. Muscle-derived exosomes disseminate systemically to the liver, lungs, and spleen, with exosomes-encapsulated miR-181d-5p ameliorating metabolic dysfunction-associated steatohepatitis (MASH) [53]. Notably, pancreatic β-cell-specific overexpression of mutant miR-29a results in significantly higher hepatic accumulation compared with skeletal muscle and adipose tissue [54]. BAT transplantation studies show that adipose-specific Dicer knockout (ADicerKO) mice exhibit approximately 50% reduced hepatic FGF21 expression [19]. Collectively, these observations raise the fundamental question of whether extracellular vesicle (EV)-mediated inter-tissue crosstalk occurs through universal membrane fusion mechanisms or exhibits tissue-specific recognition systems. To address this, we performed proteomic profiling of exosomes isolated from white and brown adipocytes to identify BAT-derived exosome-specific surface membrane proteins. Building on these findings, our ongoing work aims to elucidate the molecular mechanisms governing the hepatic targeting specificity of BAT-derived exosomes.

PPP, a branch of glycolysis, involves three key enzymes: G6PD, 6-phosphogluconate dehydrogenase (PGD), and TKT. The oxidative branch (G6PD and PGD) generates NADPH and ribulose-5-phosphate (Ru-5-P) for macromolecule biosynthesis, while the non-oxidative branch (TKT) interconverts carbohydrates to supply biomolecule synthesis. G6PD deficiency increases erythrocyte oxidative susceptibility [55], and treatment of cells (e.g. hepatocytes, HUVECs, CD4^+^ T, and CD8^+^ T cells) with G6PD inhibitors reduces NADPH levels [56]. PGD mediates NADPH and Ru-5-P production, serving as precursors for nucleotide biosynthesis and lipogenesis [57, 58]. Liver-specific *Tkt* knockout elevates R-5-P and nucleotides (GMP/IMP/CMP), promoting DNA synthesis while attenuating lipid accumulation [59, 60]. Although these studies focus on individual enzymes, the systemic consequences of PPP inhibition *in vivo* remain unclear. We demonstrate that hepatic miR-206-3p targets PPP enzymes (G6PD and TKT), thereby reducing NADPH levels and accumulating lipogenic substrates (acetyl-CoA and glycerol-3-phosphate), while concomitantly downregulating lipogenic genes. This establishes a mechanistic link between PPP suppression and attenuated lipogenesis. Additionally, reduced NADPH contributes to decreased serum AST and ALT levels. Metabolic profiling in mice with liver-specific miR-206 overexpression revealed increased Ru-5-P, GMP, IMP, dGMP, and CMP, consistent with PGD or TKT knockout models [57–59]. We also observed enhanced hepatic proliferation, with RNA-seq showing enrichment of proliferation-related pathways. However, further evidence is needed to clarify the dominant roles of miR-206-3p. Overall, our findings enhance the understanding of the complex interplay among miR-206-3p, PPP, and hepatic lipid metabolism, offering valuable insights for future research.

In summary, our findings demonstrate that BAT-derived exosomal miR-206-3p traffics to the liver and ameliorates hepatic steatosis by suppressing PPP, identifying a novel therapeutic target for obesity-induced hepatic steatosis.

### Limitations of the study

Our study confirms that BAT-derived exosomal miR-206-3p functions in the liver. However, additional *in vivo* experiments are required to determine whether BAT-secreted exosomes are specifically taken up by the liver and whether miR-206-3p mediates this process. We also found that miR-206-3p alleviates hepatic lipid accumulation by targeting the PPP to inhibit NADPH production. This inhibition is accompanied by the accumulation of acetyl-CoA and glycerol-3-phosphate (substrates for lipid synthesis) along with suppressed expression of genes involved in *de novo* lipogenesis. Nevertheless, further experimental evidence is needed to establish the relationship between PPP and *de novo* lipogenesis.

## Materials and methods

### Animals

All mice were maintained under standard laboratory conditions (12-h light/12-h dark cycle, 23°C) with *ad libitum* access to food and water. Mice were randomly assigned to experimental groups. All procedures complied with the guidelines of the Fudan University Shanghai Medical College Animal Care and Use Committee (20230301-043) and the National Institutes of Health (NIH) standards for laboratory animal use.

Male *ob/ob*, *db/db*, and WT C57BL/6J mice were obtained from GemPharmatech Co., Ltd. at 8 weeks of age. For diet-induced obesity models, 8-week-old C57BL/6J mice were fed HFD (60% kcal fat, D12492; Research Diets) for 16 weeks.

The miR-206^flox/flox^ mice were generated on a C57BL/6J background using CRISPR/Cas9-mediated insertion of LoxP sites flanking the miR-206 locus. Adipose-specific knockout mice were created by crossing miR-206^flox/flox^ mice with *Adipoq*-Cre transgenic strains.

### Human samples

Serum samples were collected from individuals undergoing routine physical examinations and classified into healthy controls and patients with MAFLD based on B-mode ultrasonography. Liver biopsy specimens were collected from patients with liver diseases and categorized into normal controls and patients with MAFLD based on histopathological evaluation of H&E staining. Human SVF was isolated from abdominal subcutaneous adipose tissue. The study was conducted in accordance with the Declaration of Helsinki and approved by the ethics committee of Zhejiang Sian International Hospital (XA-K-2023-010).

### Isolation and characterization of adipose tissue-derived exosomes

Fresh BAT from WT mice and *ob/ob* mice was dissected into fragments smaller than 0.1 cm^3^. Fragments were cultured in Dulbecco’s modified Eagle medium (DMEM)/F-12 medium supplemented with 10% exosome-depleted fetal bovine serum (FBS), 1% penicillin−streptomycin, and 1% biotin for 72 h. Supernatants were collected every 36 h with medium replenishment. A total of 20 mL collected supernatants were centrifuged at 3,000 *g* for 15 min (4°C) to remove debris and cells. Exosomes were isolated by ultracentrifugation after filtration through a 0.22-μm membrane. Briefly, samples were centrifuged at 120,000 *g* for 1 h at 4°C (Optima XE-90 ultracentrifuge, Type 41 Ti rotor, Beckman Coulter), and the supernatant was discarded. The pelleted exosomes were resuspended in PBS and centrifuged again at 120,000 *g* for 1 h at 4°C to obtain purified exosomes.

The exosomes diluted in PBS were subjected to NanoSight (Malvern Panalytical, UK) for NTA. The exosome morphology was examined by transmission electron microscope (Tecnai G2 Spirit, FEI Corp. USA) at Fudan University Electron Microscopy Center. This ultracentrifugation method is suitable for the isolation of exosomes from tissues and cell culture supernatants.

### Serum exosome purification and characterization

Serum samples were thawed and centrifuged at 3,000 *g* for 15 min at 4°C to remove residual cell debris. Totally, 250 μL of each supernatant were transferred into a clean 1.5-mL Eppendorf tube and incubated with pre-warmed thromboplastin D (Sigma, 44213-1V) at 37°C for 15 min. After centrifugation at 10,000 *g* for 5 min at room temperature, the supernatants were transferred to fresh tubes for exosome isolation.

Exosomes were isolated using ExoQuick Exosome Precipitation Solution (SBl, Cat#:100356EX0Q20A-1, Mountain View, CA) mixed with ribonuclease A (RNase A, Sigma, Cat# R6513-10MG) at a final concentration of 10 μg/mL. The mixtures were incubated at 4°C for 12 h, followed by the addition of 150 U/mL murine RNase inhibitor (NEB, Cat# M0314L). Exosomes were precipitated by centrifugation at 1,500 *g* for 30 min at room temperature. The resulting exosome pellets were washed and resuspended in 25 µL sterile PBS. Exosomal miRNA was then isolated using the miRNeasy Micro Kit (QlAGEN, Cat# 217084) according to the manufacturer’s protocol. This method is suitable for the isolation of exosomes from both human and mouse serum samples.

### miRNA sequencing and RT-qPCR

Total RNA was extracted using TRIzol (Invitrogen), and then dissolved in DEPC-treated H_2_O and stored at −20°C. Libraries were prepared with the NEBNext Multiplex Small RNA Library Prep Kit for Illumina (Illumina, USA) and sequenced on the Illumina HiSeq platform. Bioinformatics analysis identified 6185 small RNAs with fragments per kilobase of transcript per million mapped reads (FPKM) ≥ 1. Differential expression analysis (fold-change cutoff: 2.0) revealed 71 up-regulated and 109 down-regulated miRNAs in the *ob/ob* group.

The miRNA was synthesized from total RNA using the miRNA 1st Strand cDNA Synthesis Kit (by stem-loop) (Vazyme, MR101-01). The mRNA was synthesized using the Maxima H Minus First-Strand cDNA Synthesis Kit with dsDNase (Thermo Fisher, K1682). The cDNA was then amplified using SYBR Green PCR Master Mix (Vazyme) with ViiA^TM^ 7 Real-Time PCR System (Applied Biosystems). The miRNA levels were normalized to *U6* snRNA using the 2^−ΔΔCT^ method, and the results were expressed as fold changes relative to the control group. The primers used are listed in Table 3.

**Table 3.** Primers for RT-qPCR used in this study.

Genes	Forward (5′−3′)	Reverse (5′−3′)
*U6*	CTCGCTTCGGCAGCACA	AACGCTTCACGAATTTGCGT
*miR-206-3p*	GCGCGTGGAATGTAAGGAAGT	AGTGCAGGGTCCGAGGTATT
*18s*	CGCCGCTAGAGGTGAAATTCT	CATTCTTGGCAAATGCTTTCG
*G6pd*	CACAGTGGACGACATCCGAAA	AGCTACATAGGAATTACGGGCAA
*Pgd*	ATGGCCCAAGCTGACATTG	GCACAGACCACAAATCCATGAT
*Tkt*	TGCACGCCATAATCAACCCTG	CATGCACTCACTTTTGCAGTTT
*Ucp1*	ATCAGGGTATCCTCTCCCCAG	CTGAGTGAGGCAAAGCTGATTT
*Cidea*	TGACATTCATGGGATTGCAGAC	GGCCAGTTGTGATGACTAAGAC
*Pgc1α*	TATGGAGTGACATAGAGTGTGCT	CCACTTCAATCCACCCAGAAAG
*Prdm16*	CCACCAGCGAGGACTTCAC	GGAGGACTCTCGTAGCTCGAA
*Pparγ*	ACCAAAGTGCAATCAAAGTGGA	ATGAGGGAGTTGGAAGGCTCT
*Fabp4*	GGGGCCAGGCTTCTATTCC	GGAGCTGGGTTAGGTATGGG
*Srebp1c*	GGAGCCATGGATTGCACATT	GGCCCGGGAAGTCACTGT
*Acc*	TGTACAAGCAGTGTGGGCTGGCT	CCACATGGCCTGGCTTGGAGGG
*Fasn*	GGAGGTGGTGATAGCCGGTAT	TGGGTAATCCATAGAGCCCAG

### Cell culture and induction of differentiation

All cell lines were cultured in their respective media at 37°C and 5% CO_2_. The primary brown adipocytes were cultured in DMEM (Gibco, Cat#11995) supplemented with 10% FBS (Gibco, Gaithersburg, MD, USA). Upon reaching 80% confluence (designated as day −2), the cells were treated with differentiation medium (DMEM with 10% FBS, 10 μg/mL insulin, and 2 nmol/L triiodothyronine) for 2 days. From day 0 through day 2, cells were exposed to induction medium (DMEM with 10% FBS, 10 μg/mL insulin, 2 nmol/L triiodothyronine, 0.5 mmol/L 3-isobutyl-1-methylxanthine, 1 μmol/L dexamethasone, and 0.125 mmol/L indomethacin). The medium was replaced with differentiation medium from day 2 to day 4, followed by maintenance in DMEM with 10% FBS until day 8. HEK293T and HEK293A cells were cultured in DMEM supplemented with 10% FBS under standard conditions.

### Isolation of primary hepatocytes

After the mice were anesthetized, an abdominal incision was made to expose the liver, the renal vein was clamped, and a catheter was inserted into the portal vein. The infrahepatic vena cava was severed, and D-Hanks buffer was perfused through the portal vein until the liver turned pale. Subsequently, 0.08% Type IV collagenase was perfused to digest the liver until the tissue turned pink, and then stopped. The intact liver was then washed in D-Hanks buffer and transferred to DMEM. The liver surface membrane was gently peeled off with forceps to release hepatocytes. The cell suspension was filtered through a 70-μm mesh, and live/dead cells were separated by 45% Percoll gradient centrifugation. Isolated live cells were washed twice with DMEM and collected by centrifugation at 50 *g* for 5 min, and then resuspended in DMEM complete medium for subsequent experiments.

### Transfection of mimics/inhibitors

Commercially available miRNA mimics or inhibitors, along with NC mimics or inhibitors (RiboBio, Guangzhou, China), were used for the functional analysis. Hepatocytes or HEK293T cells were seeded at 80% confluency in 12-well plates and transfected with miRNA mimics (25 nmol/L)/inhibitors (50 nmol/L) using RNAiMAX (Invitrogen). The medium was replaced with fresh medium 24 h after transfection. Cells were harvested 48 h after transfection for western blotting or RNA analysis. For adipocyte experiments, cells were transfected on day −3 and cultured until reaching maturity. The culture supernatants were then collected for either exosome isolation or subsequent treatment of hepatocytes.

miR-206-3p mimic:

5′-UGGAAUGUAAGGAAGUGUGUGG-3′ (Sense);

5′-CCACACACUUCCUUACAUUCCA-3′ (Antisense).

NC mimic:

5′-UUUGUACUACACAAAAGUACUG-3′ (Sense);

5′-CAGUACUUUUGUGUAGUACAAA-3′ (Antisense).

miR-206-3p inhibitor:

5′-CCACACACUUCCUUACAUUCCA-3′ (Sense);

NC inhibitor: 5′-CAGUACUUUUGUGUAGUACAAA -3′ (Sense).

### Co-culture and supernatant treatment experiments

Co-culture experiments were performed using 12-well Transwell plates with 0.4-μm pore-sized filters (Corning Costar, USA) for 24 h. Mature adipocytes were seeded in the Transwell inserts, while primary hepatocytes were seeded in the bottom chamber.

The supernatant from adipocytes transfected with miRNA mimics or inhibitors was used to treat primary hepatocytes for 24 h, followed by incubation in fresh DMEM medium (supplemented with 10% FBS) for an additional 24 h before RNA detection.

### miRNASCOPE

Fresh tissue samples were fixed in 10% formalin for 24 h, then dehydrated, cleared, embedded, and sectioned to obtain paraffin-embedded sections. To preserve RNA integrity, all samples were processed for *in situ* hybridization within one month of paraffin embedding.

All experimental procedures followed the manufacturer’s protocols (Advanced Cell Diagnostics). For BAT, antigen retrieval was performed for 15 min, followed by 30 min of proteinase treatment.

### *In vivo* and *in vitro* exosome trafficking assays

To label exosomes with PKH26 dye, 6 μL PKH26 dye was dissolved in 1 mL Solution C, and ultracentrifugation-purified exosomes were resuspended in 1 mL Solution C. The solutions were gently mixed and incubated at room temperature for 5 min. The reaction was terminated by adding 2 mL PBS with 10% BSA, followed by exosome collection via ultracentrifugation.

For *in vivo* tracking, mice were intravenously injected with 20 μg PKH26-labeled exosomes. After 24 h, exosome distribution was analyzed using an *in vivo* imaging system (IVIS). For *in vitro* studies, primary hepatocytes were treated with 5 μg PKH26-labeled exosomes for 24 h, and then fixed with 4% paraformaldehyde for 15 min and co-stained with DAPI (4’-6-diamidino-2-phenylindole) and phalloidin.

### Western blot analysis

Western blot analyses were performed as previously described. Primary antibodies against the following proteins were used: CD9 (Santa Cruz, sc-13118, 1:200), TSG101 (ProteinTech Group, 28283-1-AP, 1:200), ALIX (ProteinTech Group, 12422-1-AP, 1:1000), peroxisome proliferator-activated receptor gamma (PPARγ, CST, #2443, 1:1000), G6PD (Abcam, ab210702, 1:1000), PGD (Abcam, ab12199, 1:1000), TKT (Santa Cruz, sc-390179, 1:500), and HSP90 (Santa Cruz, sc-13119, 1:1000).

During the experiment, iWAT, gWAT, and BAT were collected from the same WT mice and cultured in equal volumes of medium, and equal volumes of tissue culture supernatant were subsequently collected for exosome isolation via ultracentrifugation. The pelleted exosomes were resuspended in a standardized volume (100 µL PBS) to ensure consistent handling. For protein marker analysis, equal volumes of these protein samples were loaded to enable direct comparison of exosome secretion levels across tissues. This methodology was designed to approximate physiological conditions in mice while maintaining experimental uniformity.

To assess the exosome secretory capacity of BAT in WT versus *ob/ob* mice, we collected BAT from individual *ob/ob* mice and matched it to pooled samples from 2 to 3 WT mice to ensure equivalent starting tissue mass between groups. Following this normalization, identical *in vitro* culture conditions were applied to both groups. Exosome secretion was subsequently quantified by western blot analysis of exosomal markers.

### Glucose tolerance test (GTT)

After 8 weeks of HFD feeding, GTT was performed on the mice. Mice were fasted for 16 h before testing. After fasting, tail vein blood samples were collected to measure fasting blood glucose levels, which were recorded as the baseline at 0 min. The mice were then weighed and administered intraperitoneal glucose injections at a dose of 2 mg/g based on body weight. Blood glucose levels were monitored at 15, 30, 60, 90, and 120 min after glucose injection to assess glucose tolerance.

### Insulin tolerance test (ITT)

After 8 weeks of HFD feeding, ITT was performed on the mice. Following a 4-h fast, tail vein blood was collected to measure fasting blood glucose levels (recorded as 0 min). Mice were then weighed and administered intraperitoneal injections of insulin (0.75 mU/g body weight). Blood glucose levels were monitored at 15, 30, 60, 90, and 120 min after injection to evaluate insulin sensitivity.

### Construction of adenoviral expression vectors and infection

The adenoviral expression vector pAd/CMV/V5-DEST (Invitrogen, Carlsbad, CA, USA) encoding miR-206 was constructed according to the manufacturer’s protocol, using GFP recombinant adenovirus as the negative control. Adenovirus vectors were amplified and purified using Sartorius Adenovirus Purification kits. The miR-206 sequence was amplified with the following primers: 5′-ATACTCGAGATGAAGTCAGGTCCCAGAGATTCTT-3′ (forward) and 5′-ATAGAATTCTGGGGAAGAGGGCACCTGC-3′ (reverse). For *in vivo* studies, adenoviruses at a dose of 1 × 10^9^ plaque-forming units (PFU) per mouse were diluted in 125 μL PBS and administered via either the BAT or tail vein once weekly for 4 weeks.

### Metabolomics

Fresh liver samples (50 mg) were homogenized and extracted with 500 µL pre-cooled 70% methanol/water (−20°C). The samples were centrifuged at 2,500 rpm for 5 min, and then incubated for 5 min. This procedure was repeated twice. Subsequently, the samples were centrifuged at 12,000 rpm for 5 min at 4°C. The supernatant (400 µL) was carefully transferred to a new centrifuge tube and kept at −20°C for 30 min. Afterward, the samples were centrifuged again at 15,000 rpm for 20 min at 4°C. Finally, 200 µL of the supernatant was collected for subsequent analysis.

The analysis was performed using an ultra-performance liquid chromatography (UPLC) system (ExionLC AD) coupled with a tandem mass spectrometry (MS/MS) (QTRAP 6500+). The mass spectrometry data were qualitatively analyzed against the Metware Database (MWDB), which was constructed using standard compounds.

### Assay of NADP^+^/NADPH ratio

After treatment, cultured primary hepatocytes were collected. The NADP^+^/NADPH ratio was measured using a NADP^+^/NADPH Assay Kit (Abcam, ab65349) according to the manufacturer’s protocol.

### Oil Red O staining

The culture medium was removed from the adipocytes differentiated for 8 days. The cells were washed three times with PBS and then fixed with 4% paraformaldehyde for 15 min. After fixation, any residual paraformaldehyde was rinsed off, the Oil Red O working solution was added, and the samples were stained for 2 h at room temperature.

### Luciferase reporter assays

The *G6pd* and *Tkt* luciferase reporter plasmids (pmiR-report luciferase, 100 ng per transfection) were transiently co-transfected with a Renilla luciferase vector (pRL, 10 ng per transfection) and miR-206-3p mimic or NC mimic into HEK293 cells. After 24 h, luciferase activity was measured using the Dual-Luciferase Reporter Assay System (Promega) and normalized to Renilla luciferase activity. Each experiment was performed in triplicate and independently repeated at least three times.

### Statistical analysis

The experimental data were analyzed using GraphPad Prism 8.0 software and presented as mean ± standard deviation (SD). The Student’s *t*-test was used for comparing two parameters, while one-way analysis of variance (ANOVA) and two-way ANOVA were employed for analyses involving multiple parameters. A significance level of *P* < 0.05 indicates statistical significance. All experiments were performed in at least three independent replicates, with representative data shown.

## Supplementary Material

loaf028_suppl_Supplementary_Figures_S1-S10

## Data Availability

The authors confirm that all the data supporting the findings of this study are available within the supplementary material and corresponding authors.
