# Supplementary material for: The brown fat-enriched exosomal miR-206-3p attenuates hepatic lipogenesis by decreasing pentose phosphate pathway
Source: Life Metab. 2025 Jul 12;4(6):loaf028. doi: 10.1093/lifemeta/loaf028 (PMC12507029; doi:10.1093/lifemeta/loaf028)
Supplement: loaf028_suppl_Supplementary_Figures_S1-S10 [file loaf028_suppl_supplementary_figures_s1-s10.docx]

Supplementary Information for

**The brown fat-enriched exosomal miR-206-3p attenuates hepatic lipogenesis by decreasing pentose phosphate pathway**

Li-Jie Yang^1,‡^, Qiu-kai Tang^2,‡^, Lei Wang^1^, Yan-Jue Song^1^, Zhen-Yu Xu^1^, Xi-Ni Ma^1^, Yang Liu^1^, Shu-Wen Qian^1^, Qi-Qun Tang^1,*^, Yan Tang ^1,*,†^

^1^Key Laboratory of Metabolism and Molecular Medicine, Ministry of Education, Department of Biochemistry and Molecular Biology of School of Basic Medical Sciences and Department of Endocrinology and Metabolism of Zhongshan Hospital, Fudan University, Shanghai 200032, China

^2^Clinical Laboratory, Zhejiang Sian International Hospital, Jiaxing, Zhejiang 314031, China

^‡^These authors contributed equally to this work.

^*^Corresponding authors. Department of Biochemistry and Molecular Biology, Fudan University Shanghai Medical College, Yi Xue Yuan Road, Shanghai 200032, China. E-mail: [yantang@fudan.edu.cn](mailto:yantang@fudan.edu.cn) (Y.T.); [qqtang@shmu.edu.cn](mailto:qqtang@shmu.edu.cn) (Q.Q.T.)

^†^Lead contact.

**This file includes:**

Supplementary Figures S1−S10


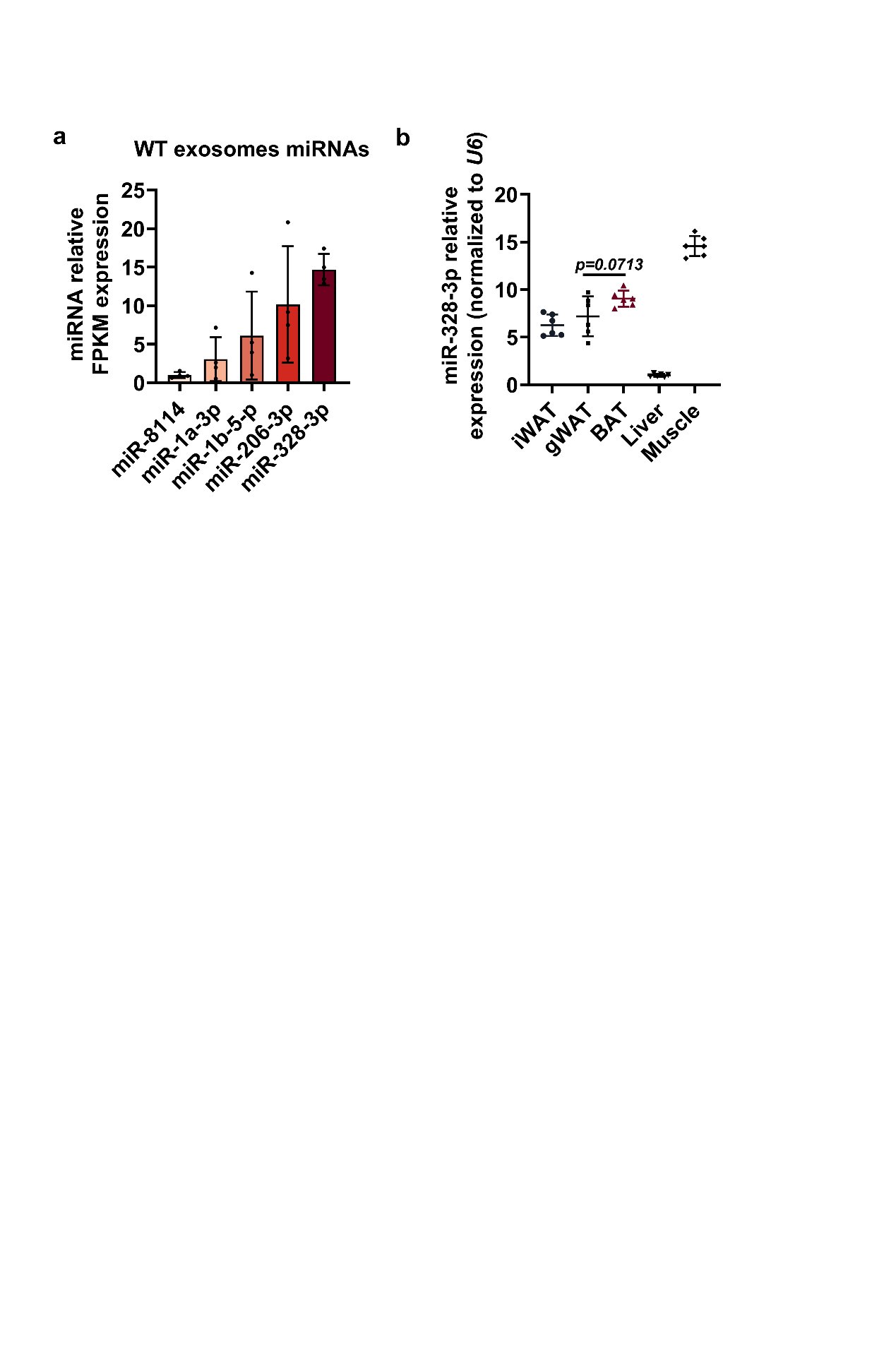
**Supplementary Figures**

**Supplementary Figure S1** The distribution of miRNAs. (a) Relative FPKM values of miR-8114, miR-1a-3p, miR-1b-5p, miR-206-3p, and miR-328-3p levels in BAT-derived exosomal miRNA sequencing from 8-week-old male C57BL/6J mice (*n* = 4). (b) qPCR analysis of relative miR-328-3p levels in iWAT, gWAT, BAT, liver, and muscle from 8-week-old male C57BL/6J mice (*n* = 6). Values are means ± SD. ^*^*P* < 0.05; ^**^*P* < 0.01; ^***^*P* < 0.001; ^****^*P* < 0.0001 by Student’s *t* test or ANOVA test.

**
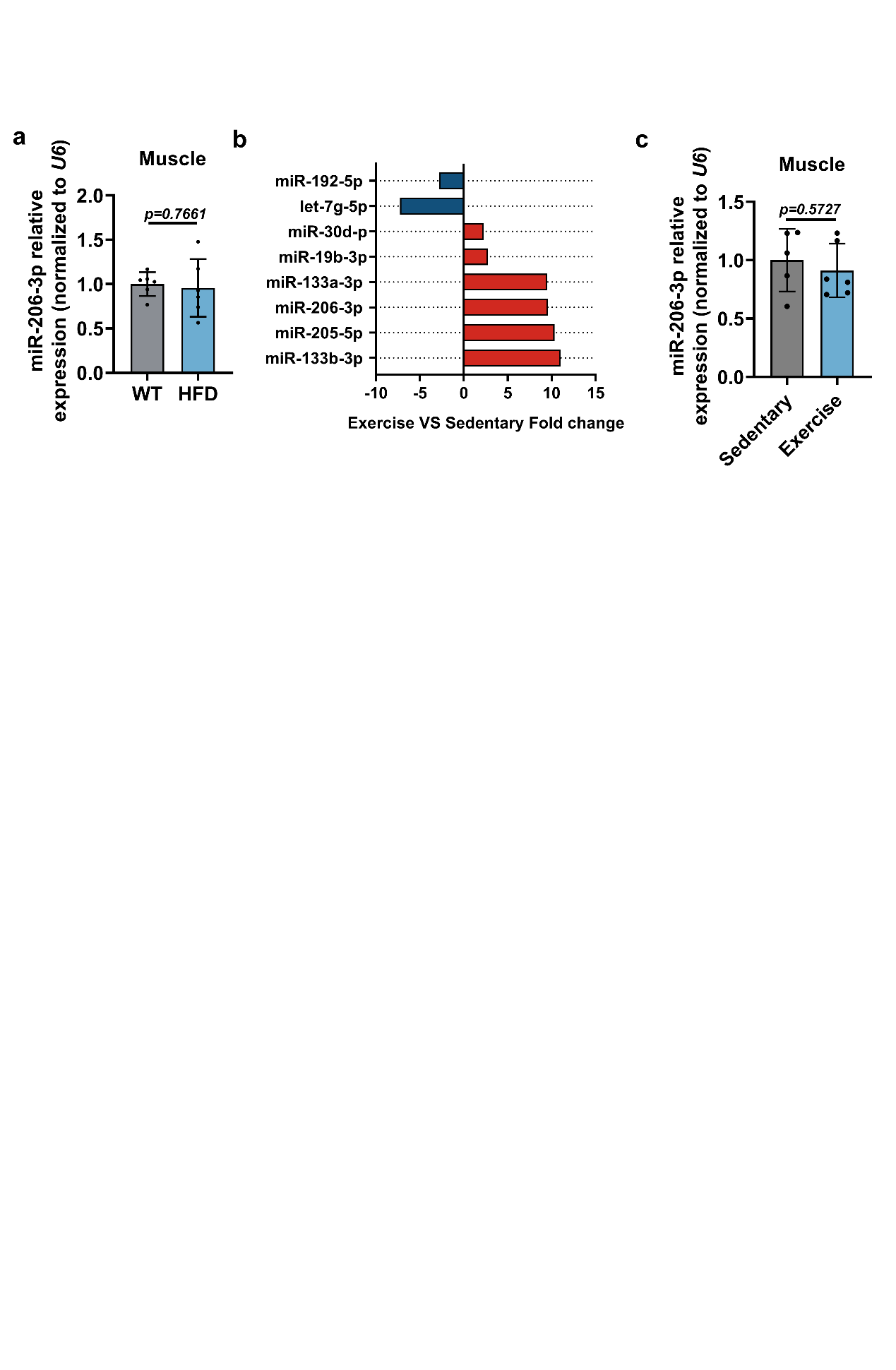
**

**Supplementary Figure S2** The expression of miR-206-3p in muscle remains stable under both physiological and pathological conditions. (a) qPCR performed to measure relative miR-206-3p levels in male C57BL/6J mice fed either ND or HFD for 16 weeks from 8 weeks of age (*n* = 6). (b) miRNA profiling results of serum-derived exosomes from sedentary and exercise-trained mice (*n* = 4). (c) qPCR analysis of relative miR-206-3p levels in muscle from sedentary and exercise mice (*n* = 6). Values are means ± SD. ^*^*P* < 0.05; ^**^*P* < 0.01; ^***^*P* < 0.001; ^****^*P* < 0.0001 by Student’s *t* test or ANOVA test.

**
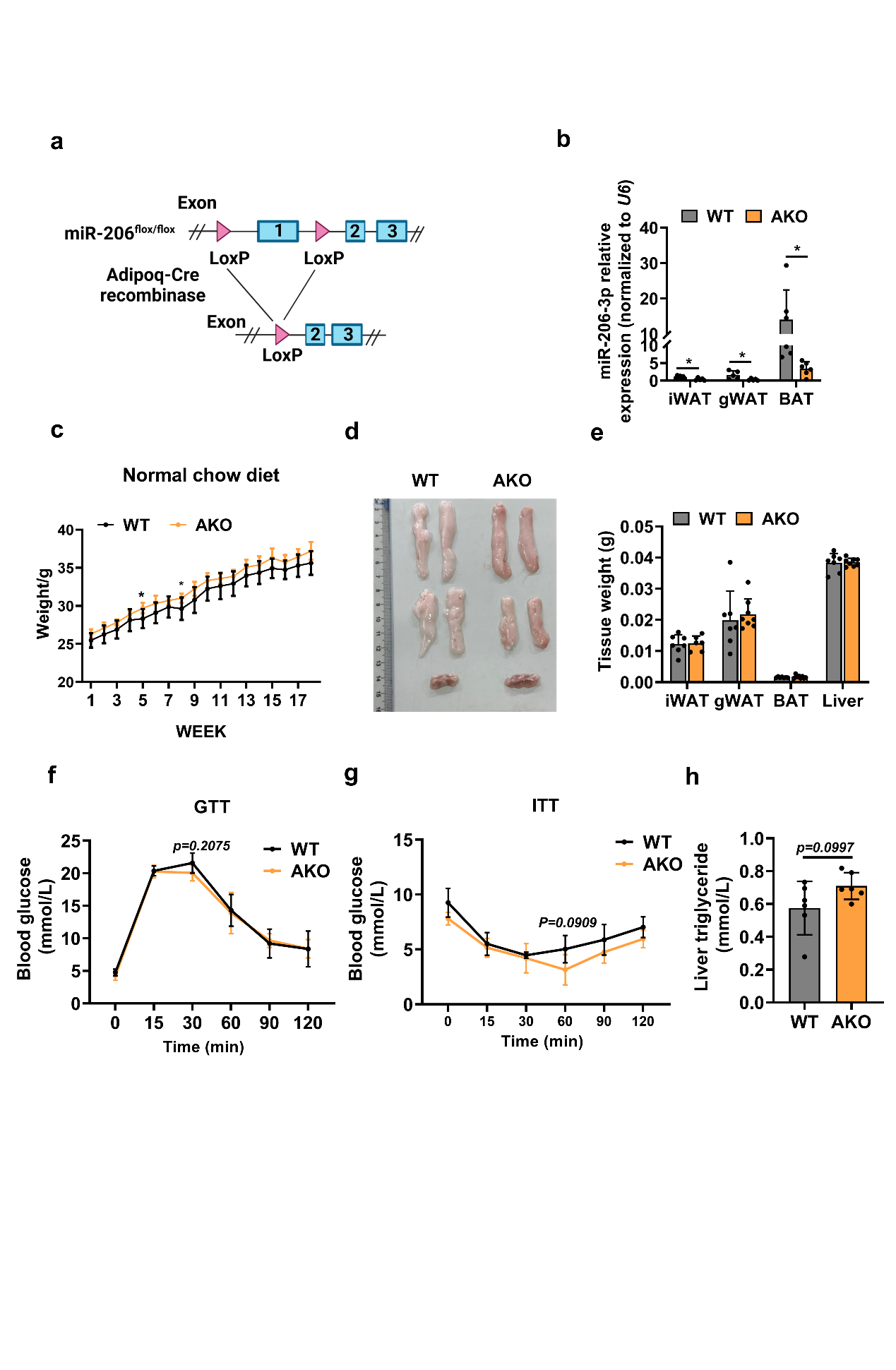
**

**Supplementary Figure S3** Generation of adipose tissue-specific miR-206 knockout (AKO) mice. (a) Schema of miR-206 AKO mouse models. (b) qPCR analysis of relative miR-206-3p levels in iWAT, gWAT, and BAT from 8-week-old WT and AKO mice (*n* = 6). (c) Body weight of WT and AKO mice from 8 weeks of age (*n* = 12). (d) Gross morphology of iWAT, gWAT, and BAT from 24-week-old WT and AKO mice. (e) The iWAT, gWAT, BAT, and the liver weights in WT and AKO mice (*n* = 7). (f) Glucose concentrations during GTT in 19-week-old mice (*n* = 6). (g) Glucose concentrations during ITT in 18-week-old mice (*n* = 6). (h) TG levels in the liver (*n* = 6). The 8-week-old WT and AKO male mice were fed ND for 16 weeks before being sacrificed for analysis (c−h). Values are means ± SD. ^*^*P* < 0.05; ^**^*P* < 0.01; ^***^*P* < 0.001; ^****^*P* < 0.0001 by Student’s *t* test or ANOVA test.


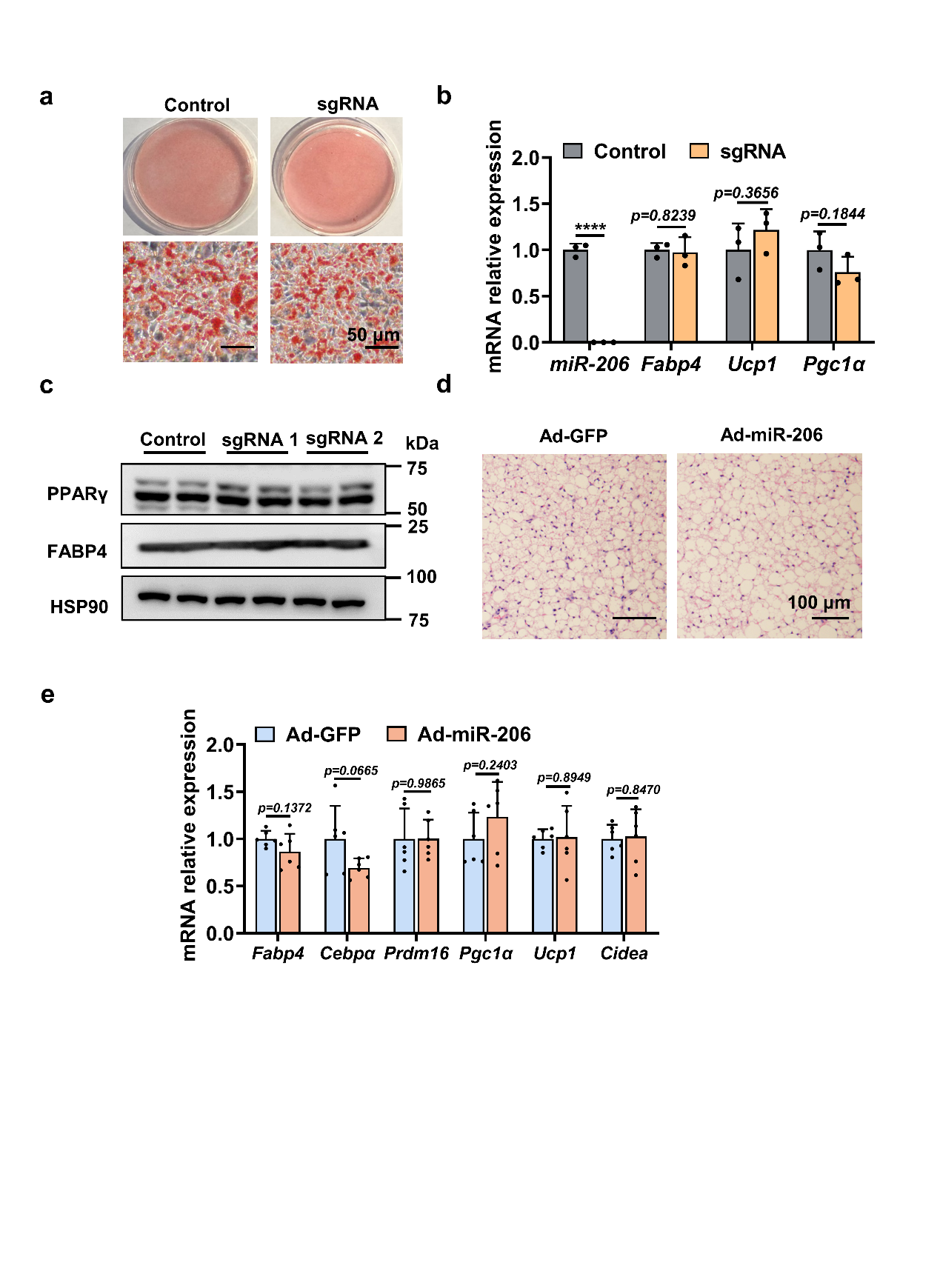


**Supplementary Figure S4** miR-206-3p does not affect brown adipocyte function. (a−c) Knockdown of miR-206-3p in pre-brown adipocytes. Crispr-Cas9-mediated knockout of miR-206-3p was generated in primary brown adipocytes. The knockout cells were then harvested on day 8 after differentiation induction for further analysis. (a) Mature adipocytes were stained with Oil Red O at day 8 to evaluate lipid accumulation. Scale bar, 50 μm. (b) Relative mRNA expression of adipogenic (*Fabp4*) and thermogenic (*Ucp1* and *Pgc1α*) genes in mature adipocytes (*n* = 3). (c) Western blot analysis of adipogenic markers PPARγ and FABP4. (d) Representative H&E staining of BAT sections. Scale bar, 100 μm. (e) Relative mRNA expression of adipogenic (*Fabp4* and *Cebpα*) and thermogenic (*Ucp1*, *Pgc1α*, *Cidea*, and *Prdm16*) genes in BAT (*n* = 6). Eight-week-old male C57BL/6J mice were fed HFD for 16 weeks and received weekly injections of Ad-GFP or Ad-miR-206 into BAT starting at week 13 (d and e). Values are means ± SD. ^*^*P* < 0.05; ^**^*P* < 0.01; ^***^*P* < 0.001; ^****^*P* < 0.0001 by Student’s *t* test or ANOVA test.


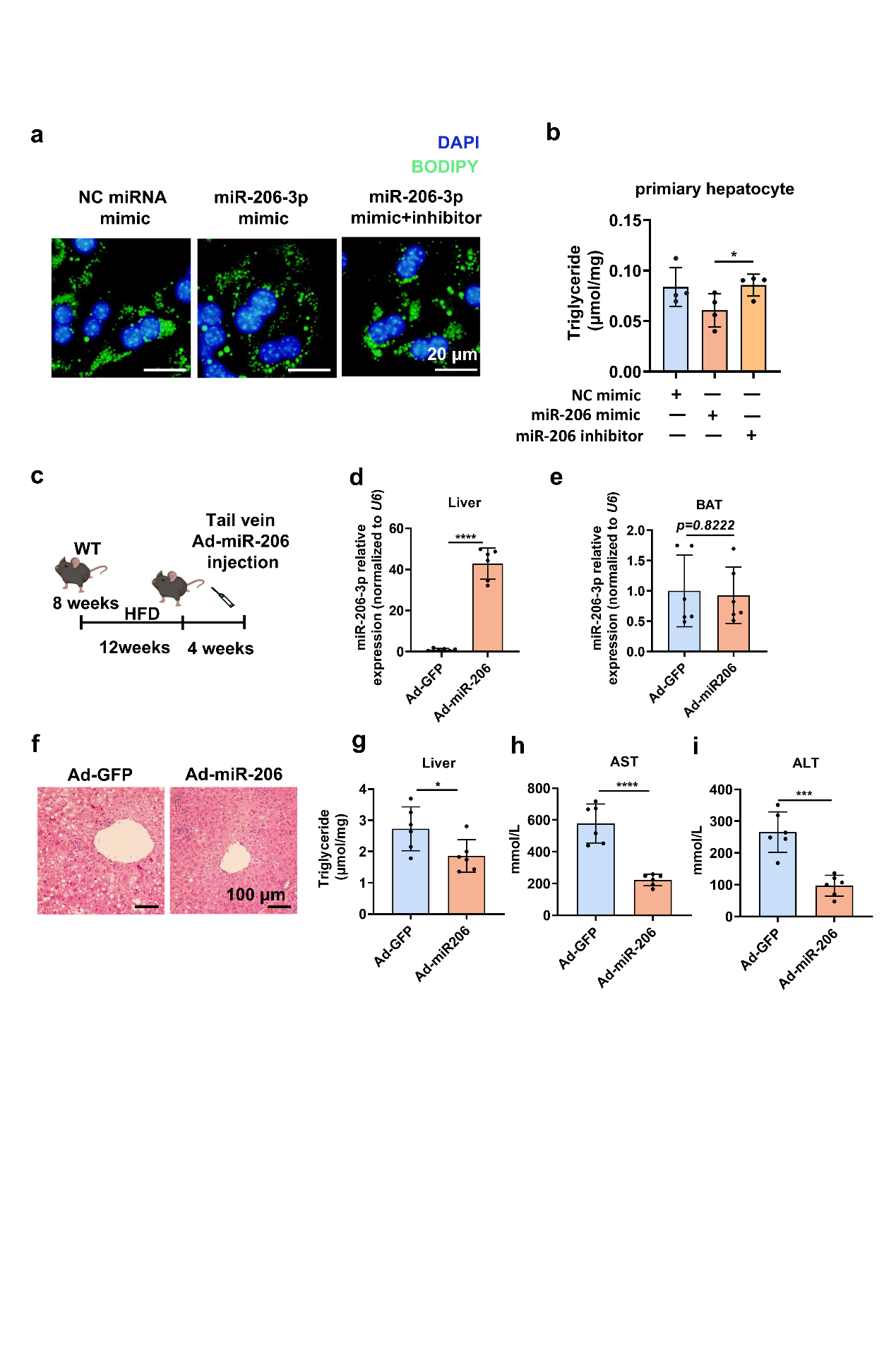


**Supplementary Figure S5** miR-206-3p can reduce hepatic lipid accumulation. (a) Representative images of BODIPY and DAPI fluorescence in primary hepatocytes. Scale bar, 20μm. (b) TG levels in primary hepatocytes (*n* = 4). Primary hepatocytes were pretreated with miR-206-3p mimic, miR-206-3p inhibitor, or NC mimic for 24 h, followed by harvesting at 24 h after treatment (a and b). (c) Schematic diagram. (d) qPCR analysis of relative miR-206-3p levels in liver (*n* = 6). (e) qPCR analysis of relative miR-206-3p levels in BAT (*n* = 6). (f) Representative H&E staining of liver sections. Scale bar, 100 μm. (g) TG levels in the liver (*n* = 6). (h) Serum AST levels in mice (*n* = 6). (i) Serum ALT levels in mice (*n* = 6). The 8-week-old male C57BL/6J mice were fed HFD for 16 weeks and received weekly tail-vein injections of Ad-GFP or Ad-miR-206 starting at week 13 (c−i) .Values are means ± SD. ^*^*P* < 0.05; ^**^*P* < 0.01; ^***^*P* < 0.001; ^****^*P* < 0.0001 by Student’s *t* test or ANOVA test.

**
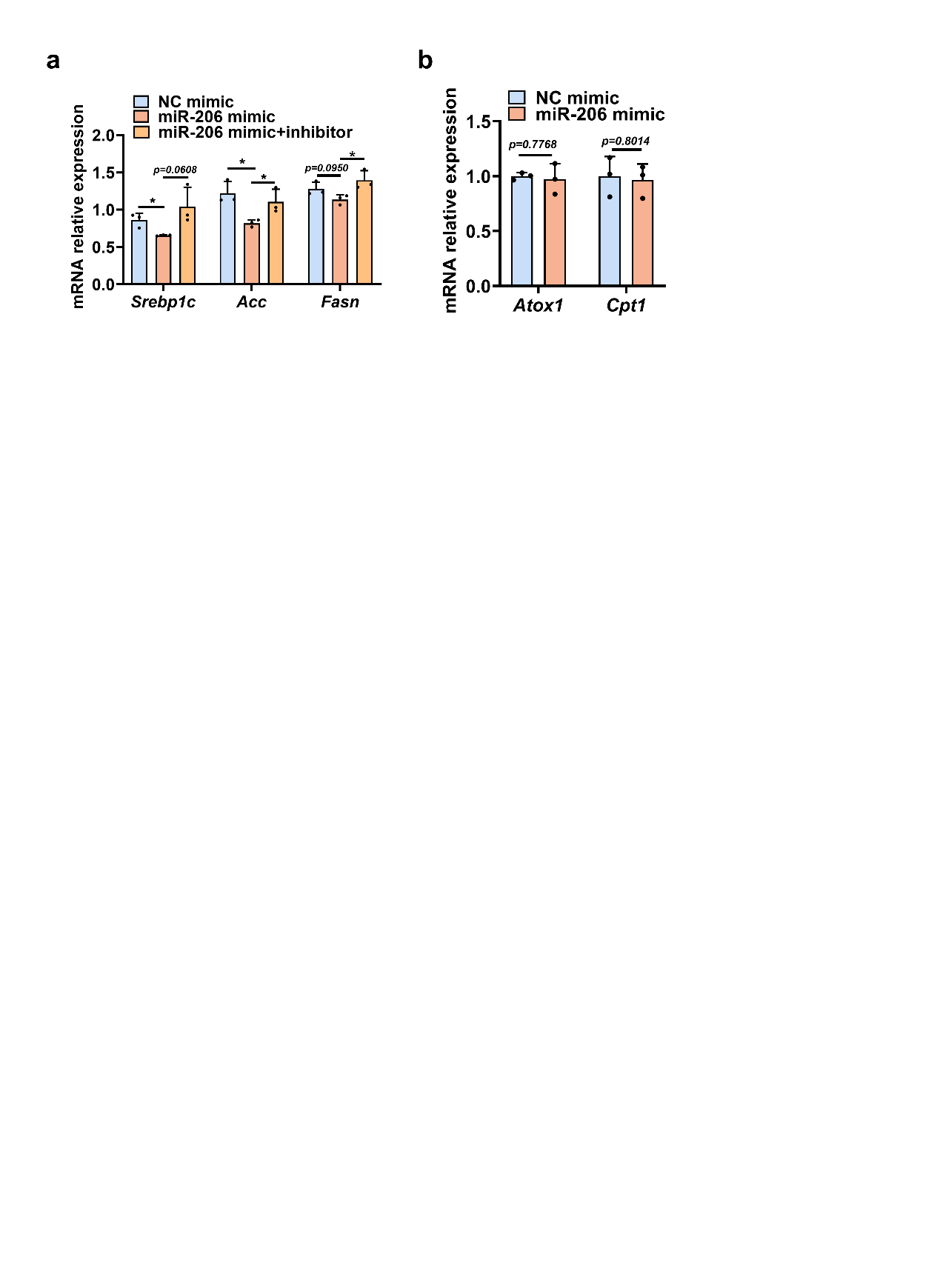
**

**Supplementary Figure S6** The pathway of *de novo* lipogenesis and β-oxidation. (a) Relative mRNA expression of *de novo* lipogenesis genes (*Srebp1c*, *Acc*, and *Fasn*) in primary hepatocytes (*n* = 3). (b) Relative mRNA expression of β-oxidation genes (*Atox1* and *Cpt1*) in primary hepatocytes (*n* = 3). Values are means ± SD. ^*^*P* < 0.05; ^**^*P* < 0.01; ^***^*P* < 0.001; ^****^*P* < 0.0001 by Student’s *t* test or ANOVA test.

**
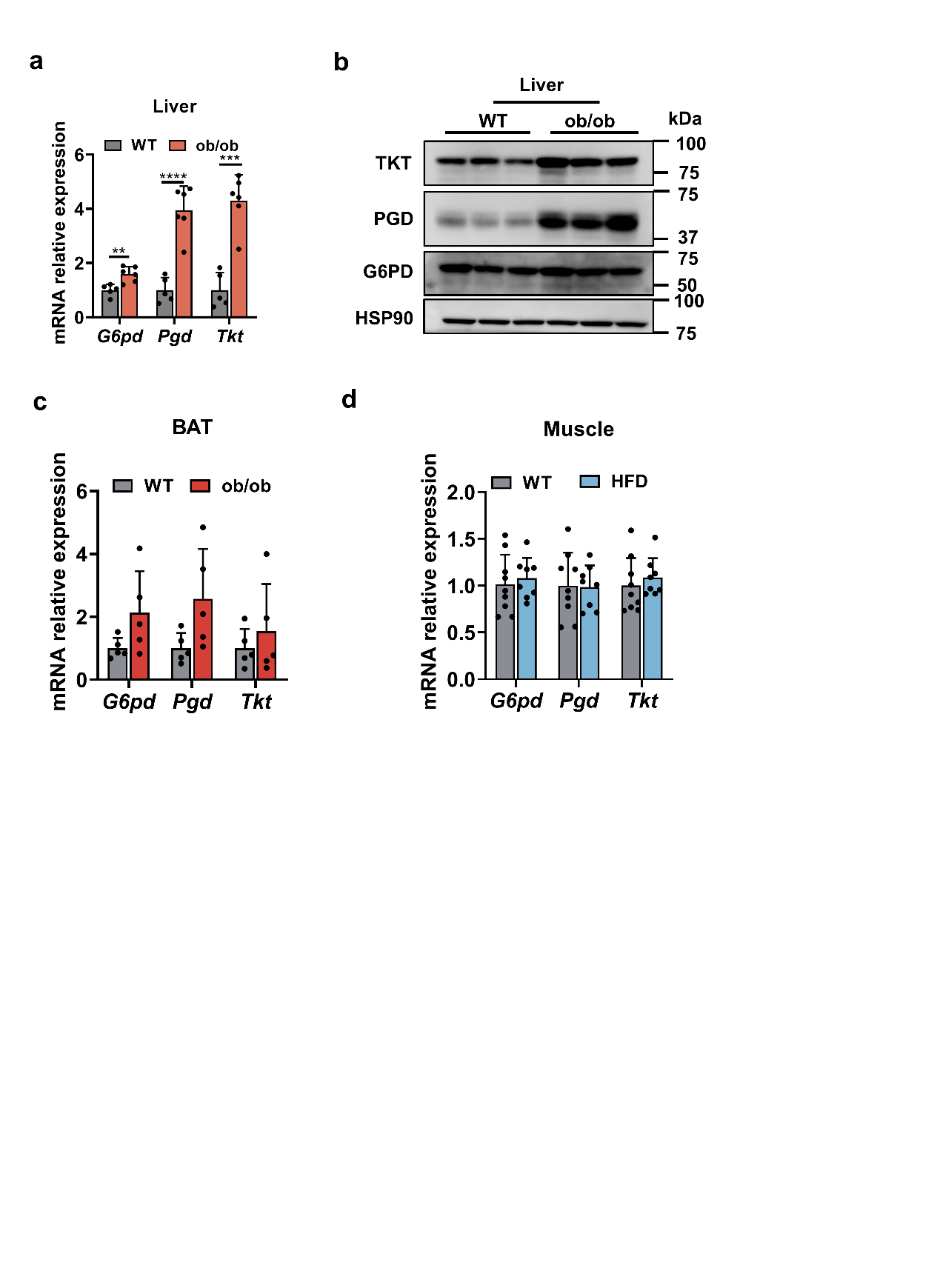
**

**Supplementary Figure S7** Characteristics of the pentose phosphate pathway (PPP). (a) Relative mRNA expression of PPP genes (*G6pd*, *Pgd*, and *Tkt*) in the liver of WT and *ob/ob* mice (*n* = 6). (b) Western blot analysis of G6PD, PGD, and TKT in the liver of WT and *ob/ob* mice. (c) Relative mRNA expression of PPP genes (*G6pd*, *Pgd*, and *Tkt*) in the BAT of WT and *ob/ob* mice (*n* = 6). (d) Relative mRNA expression of PPP genes (*G6pd*, *Pgd*, and *Tkt*) in the muscle of WT and HFD mice (*n* = 8−9). Values are means ± SD. ^*^*P* < 0.05; ^**^*P* < 0.01; ^***^*P* < 0.001; ^****^*P* < 0.0001 by Student’s *t* test or ANOVA test.

**
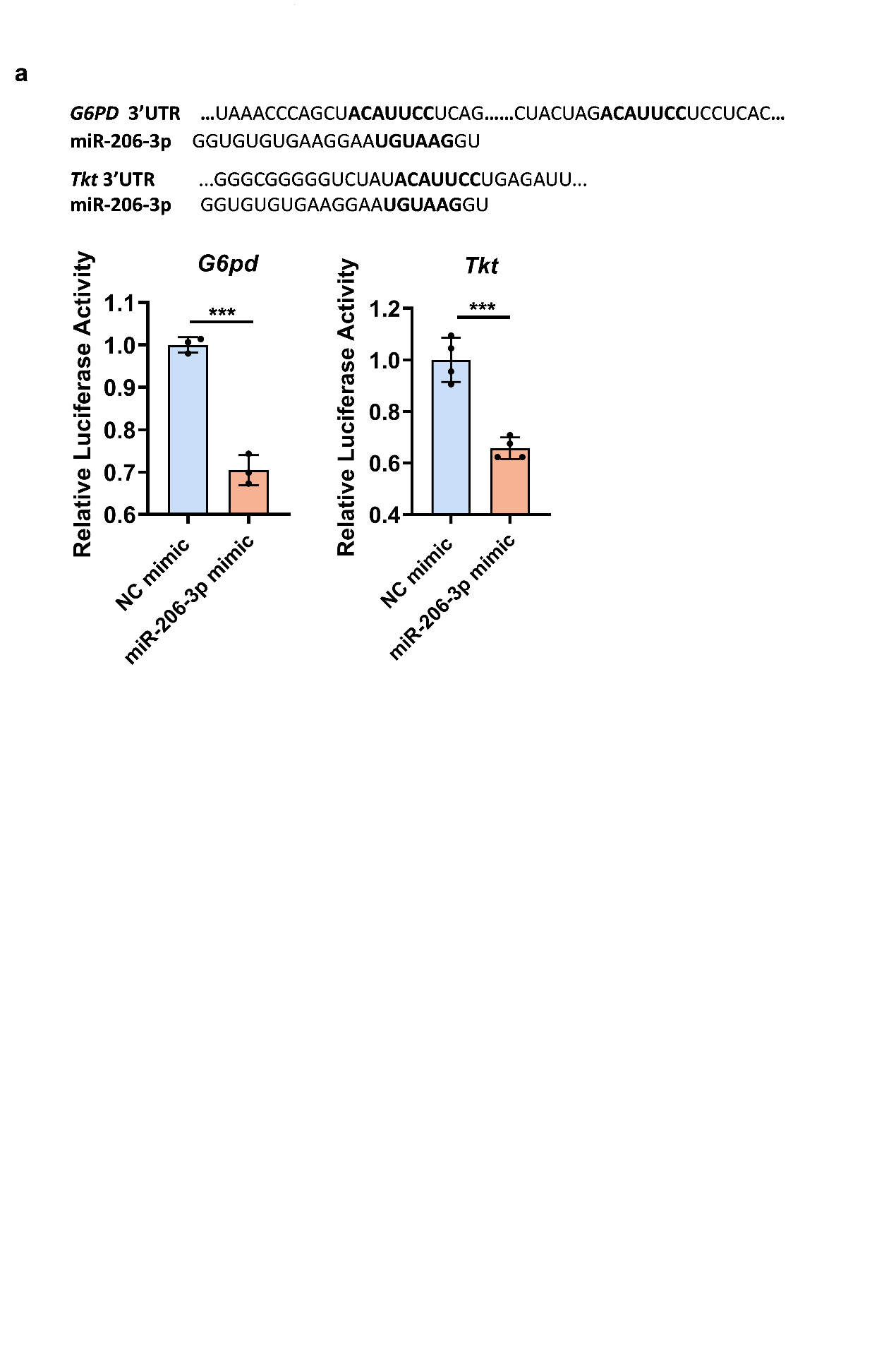
**

**Supplementary Figure S8** *G6pd* and *Tkt* are miR-206-3p target genes. Luciferase activity in 293T cells co-transfected with miR-206-3p and reporter vectors containing 3’-UTRs of *G6pd* or *Tkt* (*n* = 3−4).


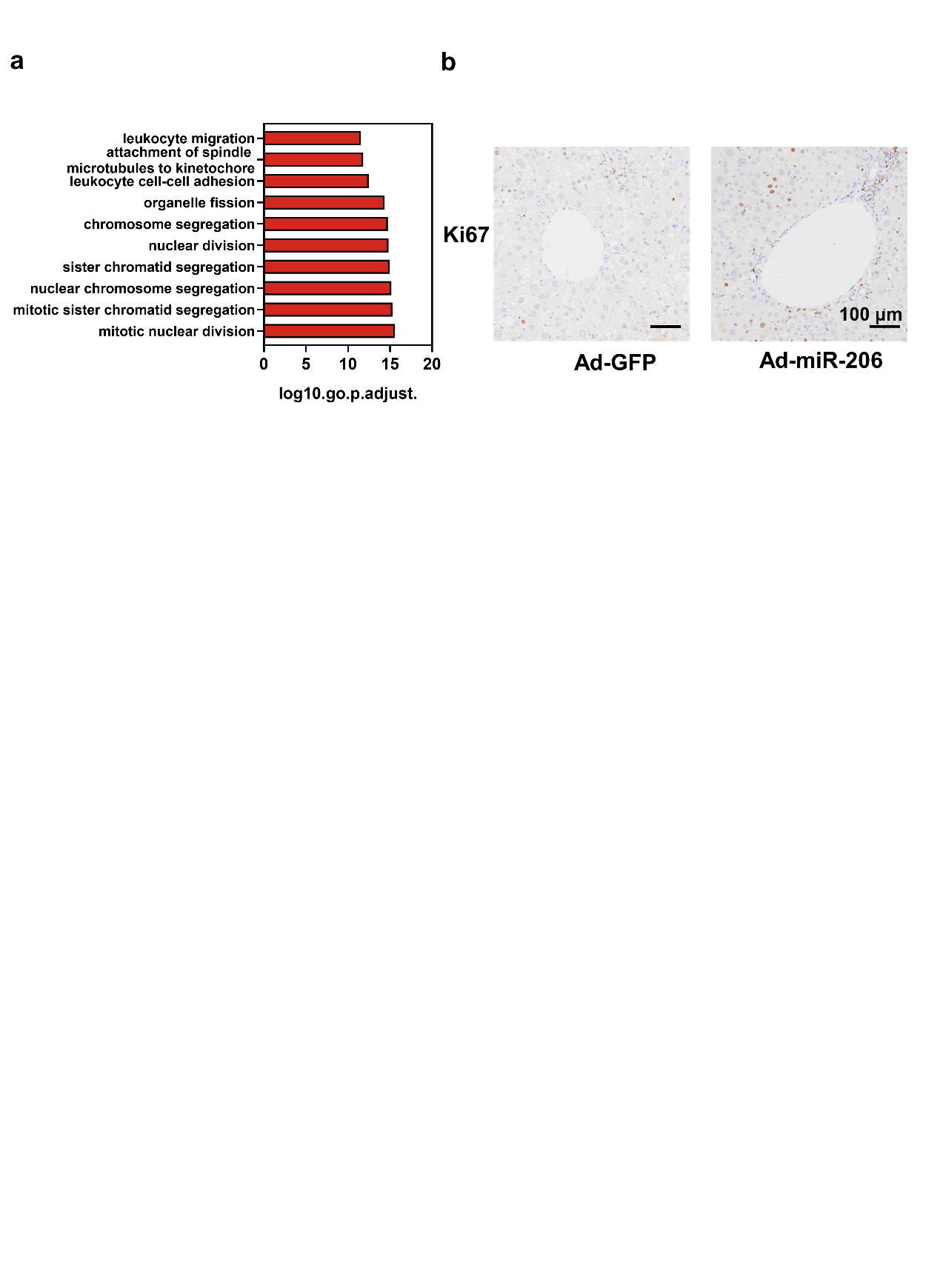


**Supplementary Figure S9** miR-206-3p promotes cell proliferation. (a) Gene Ontology (GO) enrichment analysis performed on upregulated genes, with the top 10 terms selected based on log_10_(*P* value). (b) Representative Ki67 staining patterns in liver tissue sections from Ad-GFP and Ad-miR-206-injected mice. Scale bar, 100 μm. The 8-week-old male C57BL/6J mice were fed HFD for 16 weeks and received weekly tail-vein injections of Ad-GFP or Ad-miR-206, starting from week 13 (a and b) .


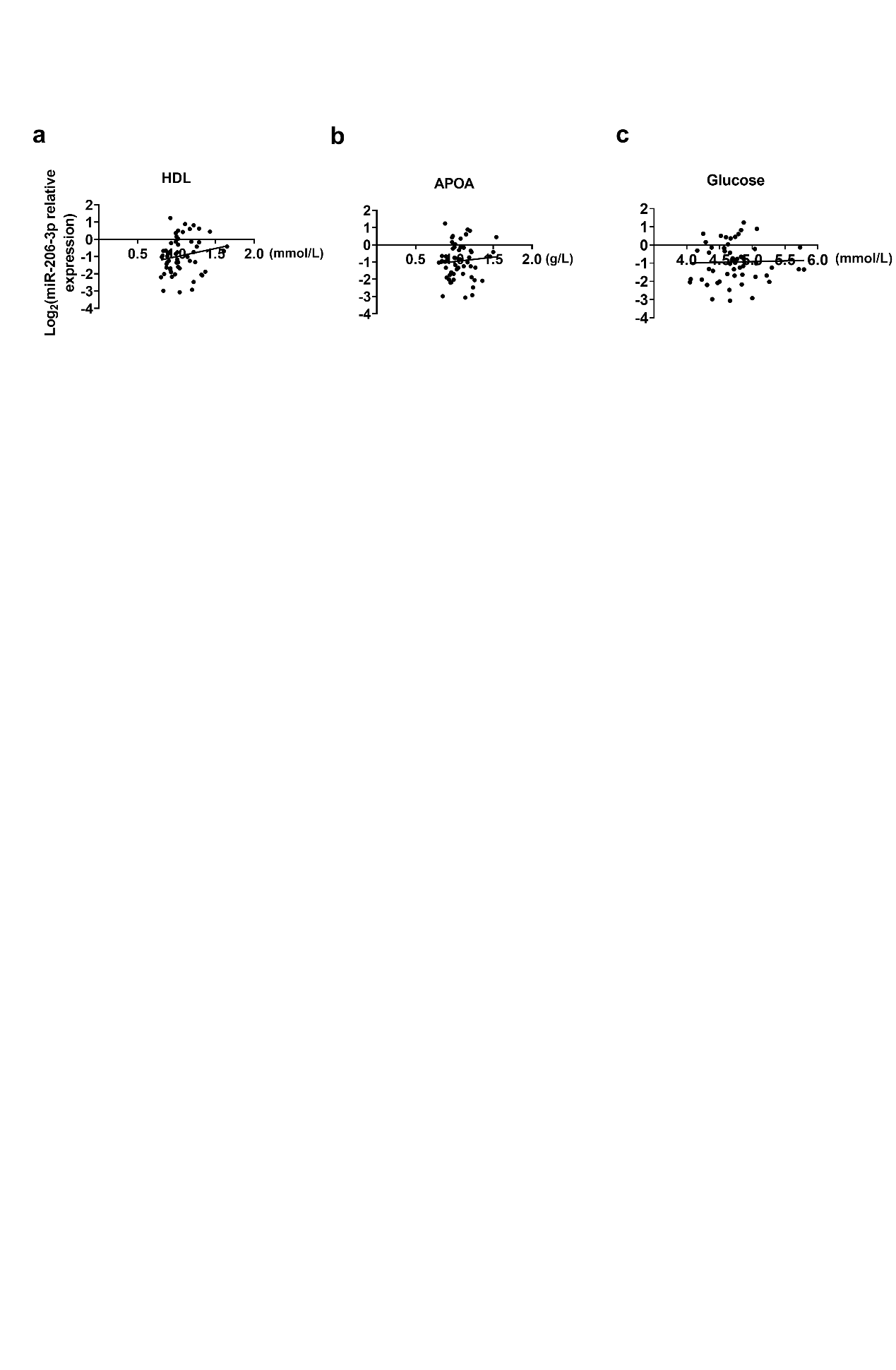


**Supplementary Figure S10** The correlaton between miR-206-3p and serum metabolic parameters. (a−c）Correlation of HDL (a), APOA (b), and glucose (c) with miR-206-3p levels in serum-derived exosomes (*n* = 50).
